# The longitudinal effect of repetition and practice on the accuracy of lay anal examinations for detecting perianal and anal canal abnormalities: a prospective study

**DOI:** 10.1016/j.lana.2025.101317

**Published:** 2025-12-06

**Authors:** Alan G. Nyitray, Timothy L. McAuliffe, Jenna Nitkowski, Cameron Liebert, Michael D. Swartz, Ashish A. Deshmukh, Jared Kerman, Ellen Almirol, John A. Schneider, J. Michael Wilkerson, Lu-Yu Hwang, Derek Smith, Duo Yu, Aniruddha Hazra, Elizabeth Y. Chiao

**Affiliations:** a Center for Cancer Discovery, Medical College of Wisconsin, Milwaukee, WI, USA; b Center for Community Health and Intervention Research, Medical College of Wisconsin, Milwaukee, WI, USA; c School of Medicine and Public Health, University of Wisconsin–Madison, Madison, WI, USA; d Department of Biostatistics and Data Science, The University of Texas Health Science Center at Houston School of Public Health, Houston, TX, USA; e Department of Public Health Sciences, Medical University of South Carolina, Charleston, SC, USA; f Cancer Control Program, Hollings Cancer Center, Medical University of South Carolina, Charleston, SC, USA; g University of Chicago, Section of Infectious Diseases and Global Health, Chicago, IL, USA; h Department of Health Promotion and Behavioral Sciences, The University of Texas Health Science Center at Houston School of Public Health, Houston, TX, USA; i Department of Epidemiology, Human Genetics, and Environmental Sciences, The University of Texas Health Science Center at Houston School of Public Health, Houston, TX, USA; j The Crofoot Research Center, Houston, TX, USA; k Data Science Institute, Medical College of Wisconsin, Milwaukee, WI, USA; l MD Anderson Cancer Center, Houston, TX, USA

**Keywords:** Anus neoplasms, Early detection of cancer, Self-examination, Digital anal rectal examination, HIV, Anal canal, Homosexuality, Male

## Abstract

**Background:**

While anal cancer screening is now recommended in several countries for high-incidence populations, barriers impede screening uptake. It is well known that patient recognition of symptoms increases cancer screening uptake, and we previously demonstrated that patients can detect small masses at the perianus and in the anal canal. In this current analysis, we aimed to longitudinally assess the effect of repetition and practice on patient self-recognition of small masses.

**Methods:**

Individuals and couples of sexual minority men and transgender women were taught to conduct an anal self-examination or anal companion examination (ASE/ACE) at visit 1 and then randomized to a practice or control condition. Six months later, at visit 2, the effect of practice and repetition of the ASE/ACE on detection of abnormalities was assessed by comparing the lay exam results to those of a clinician. Concordance, *κ,* and area under the receiver operating characteristic curves (AUC) was used to quantify the comparison.

**Findings:**

Concordance between lay exam and clinician exam increased from visit 1 (73%, 524/714) to visit 2 (95%, 535/561) (*κ* = 0.87, 95% CI 0.82–0.92). Overall AUC at visit 2 was 0.93 (95% CI 0.90–0.96). Although there was no difference in concordance between the practice (95%, 267/281) and control arms (96%, 268/280) (p = 0.69), concordance increased with ASE/ACE repetition (p_trend_ < 0.001) and was 98% (354/363) for individuals performing the ASE/ACE ≥2 times between visits. For individuals with incident abnormalities at visit 2, concordance was 100% (27/27). Results did not differ by age or HIV status. False positive and false negative results by the ASE/ACE were 2% (10/561) and 3% (16/561), respectively.

**Interpretation:**

Because lay individuals can detect anal abnormalities, clinicians conducting an anal examination may suggest that the patient's own lay exams may detect early invasive anal cancer.

**Funding:**

10.13039/100000054National Cancer Institute.

**Research in context**

**Evidence before this study**

Recommendations for squamous cell carcinoma of the anus (SCCA) screening have recently been published in several countries targeting populations with high SCCA incidence including sexual and gender minorities with and without HIV. However, many barriers to screening exist including stigma and poor infrastructure. We searched PubMed through February 16, 2025 for articles describing the diagnostic performance of lay anal examinations, using the search terms ‘anus neoplasms’, ‘anal abnormality’, ‘anal lump’, ‘anal mass’, ‘anal wart’, ‘anal condyloma’, ‘hemorrhoid’, ‘self-examination’, ‘self-screen’, ‘self-palpation’, ‘self-exploration’, and ‘self-observation’. We found our two prior publications on diagnostic accuracy of lay anal exams but no studies assessing the effect of practice on the exams' ability to detect anal abnormalities.

**Added value of this study**

This study found that over time individuals' accuracy with the lay anal exam increased to over 90%. Among 516 individuals who learned how to do the lay anal examination 6 months prior, agreement between lay exam and a clinician's exam was highest (95%) in those who performed the exam multiple times in the prior 6 months. Of 27 individuals with an incident abnormality, all detected the new lesion during the lay examination.

**Implications of all the available evidence**

Since clinicians are recommended to conduct digital anal rectal examinations when doing anal cancer screening, clinicians may advise patients during the procedure that conducting a self-examination may help them detect new abnormalities.

## Introduction

Squamous cell carcinoma of the anus (SCCA) screening is now recommended for detection of HPV-associated precancers among populations at increased risk.^1^^,^^2^ Sexual minority men with HIV in the US have an annual incidence of 85/100,000 which is 50 times higher than the incidence in the rest of the general population (1.7/100,000).^3^ In the US South and Midwest, incidence is even higher, 106/100,000 and 138/100,000, respectively.^4^ Screening is recommended for sexual minority men and transgender women with HIV starting at age 35 years and for other populations beginning at age 45 years, including sexual minority men without HIV, whose incidence exceeds 33/100,000 starting at age 45 years.^2^^,^^5^

The screening procedures recommended by the US federal government are digital anal rectal examinations (DARE), anal cytology, and HPV testing with cytology, with high-resolution anoscopy (HRA) used as a confirmatory test.^1^^,^^2^ While HRA is the gold standard for detecting precancers,^6^ DARE is recommended for palpating and/or visualizing early invasive SCCA and is especially important when HRA is not available. DARE may also be used to palpate subdermal malignant tumours that could be missed by HRA which is designed to detect squamous intraepithelial lesions that could be precancerous.^7^

While screening is recommended, uptake is limited by a low supply of skilled HRA providers.^1^^,^^2^ Screening will likely also be limited by other barriers including medical mistrust, access to health care, poor preventive healthcare uptake by males and stigmas including those associated with anal cancer, the anus, receptive anal sex, and sexually transmitted infections.^8^^,^^9^ Guidelines for SCCA screening of sexual minority men with HIV from medical organizations and state governments have been in place since at least 2007,^10^^,^^11^ yet population-based US clinical surveillance from 2019 estimated that only 8% of sexual minority men and transgender women with HIV received anal cytology in the prior year.^12^ Given individual-level barriers to anal cancer screening,^9^^,^^13^^,^^14^ we believe many individuals will need motivation to attend screening. Since lay recognition of symptoms motivates cancer screening uptake,^15^ lay recognition of abnormalities at the perianus or anal canal may encourage SCCA screening.^16^

We previously established the feasibility of self- and companion examinations for palpating and/or visualizing an abnormality at the perianus and anal canal.^17^ More recently, we observed a concordance of 73% between these lay exams and a clinician's DARE for detecting abnormalities even though these lesions were small with a median lateral dimension of 3 mm.^16^ In the current analysis, our objective was to reassess the accuracy of the lay anal exams six months after individuals were taught the procedure and then randomized to either practice or not practice the exam. We postulated that practicing the lay exam between visits would be associated with concordance between the lay exam result and DARE result at Visit 2.

## Methods

### Recruitment and enrolment

The Prevent Anal Cancer (PAC) Palpation Study (NCT04090060), based in Chicago, Illinois and Houston, Texas, USA, was a randomised clinical trial using a longitudinal protocol in each city with all individuals providing written consent for the study.

Study activities occurred in 2020–2023 with visit 1 activities detailed previously.^16^ In brief, recruitment occurred through social media, friends, in-clinic advertising, and flyers placed in lesbian, gay, bisexual, and transgender (LGBT)-friendly businesses from January 2020 to December 2022. Individuals included were aged ≥25 years, sexual minority men (cisgender or transgender) or transgender women, and reported sex with men (cis or trans) in the last five years (or identified with a minority sexual orientation). Individuals excluded had self-reported a DARE in the prior 3 months or an unresolved medical diagnosis of anal condyloma, haemorrhoids, or SCCA. Participant-facing material was provided in English in Chicago, and English and Spanish in Houston.

Interested persons completed an online eligibility survey with consents typically completed online.^18^ Individuals chose to participate as an individual or with a companion and then were scheduled for an in-person visit 1 appointment for anal self-examination/anal companion examination (ASE/ACE) instruction and assessment of exam accuracy. Most appointments in Chicago occurred at a community centre serving primarily Black sexual minority men and transgender women. In Houston, appointments occurred at a private clinic serving LGBT communities (80%) and a public HIV clinic (20%).

### Visit 1 protocol

At visit 1, physicians and advanced practice providers (APP) were trained in DARE technique which has an estimated 90% sensitivity for detecting a tumour.^7^^,^^19^ A physician in Chicago (AH) and an APP in Houston (DS) conducted a large majority of DAREs at both visit 1 and visit 2. AH is an infectious disease physician with more than 5 years’ experience specializing in sexual health care for sexual and gender minorities, including conducting DARE. Before this study, DS performed ∼30 DAREs per week for four years in a clinic specializing in infectious disease and sexual minority healthcare.

The ASE/ACE training for lay individuals only occurred at visit 1, lasted an average of 14 min,^16^ and included education about anal anatomy, anal cancer, and conducting an ASE/ACE. Most training was conducted by study staff with the remainder conducted by clinicians. Illustrated instructions taught participants to use a mirror or take a selfie to view the perianus (for ASE participants only), and to palpate the entire 360° of the anal canal. Participants were taught to insert their index finger only as deep as the second knuckle (proximal interphalangeal joint) which approximates the full length of the adult male anal canal (5 cm).^17^ Participants trained on two mannequins (Kyoto Kagaku Co., Kyoto, Japan), one with and one without a 7 mm anal canal lesion. The training goal was to teach participants to detect any perianal or anal canal abnormality, regardless of the abnormality type (e.g., wart, haemorrhoid, fissure, or tumour), using the logic that if a mass or induration, regardless of aetiology, can be palpated or seen, then malignant tumours may be recognized too. Participants were told that any abnormality they detected was very unlikely to be cancer because of cancer's rarity.

The clinician then performed a DARE on each participant according to published guidelines^7^ using the DARE as a teachable moment, e.g., “I'm feeling around your entire anal canal.” The clinician recorded the presence of abnormalities but without providing results until later in the participant's visit.

Participants were left alone in the exam room to complete the ASE or ACE and record the presence of any abnormality. Couples conducted the exam on each other after asking permission of their partner; thus, couples provided two participant results while the clinician produced two associated DARE results.

After participants received their DARE results and any required clinical referrals, they were randomized 1:1 to either a control condition or to practice. Practice consisted of performing the ASE/ACE once at home three months after visit 1 and recording the results. Practicing was not discussed with individuals in the control condition. If they enquired about practice, it was neither encouraged nor discouraged. Individuals in the practice condition were provided gloves, lubrication, and the same written instructions they used during visit 1.

### Visit 2 protocol

Staff reminded individuals in the practice arm to practice at home at the 3-month mark, midway between visit 1 and 2, and followed up to ask about ASE/ACE practice results. If the participant detected a new abnormality, staff encouraged them to come back to the site of visit 1 for a clinician's DARE. There was no communication with the control condition participants.

Before the visit 2 appointment, all participants were reminded to perform the ASE/ACE at home within 24 h of the appointment, to record their results as normal or abnormal, and to complete a pre-appointment survey. At the appointment, staff recorded the participant's home-based result which was not divulged to the clinician doing the DARE at the beginning of visit 2. AH conducted 97% of visit 2 DAREs in Chicago. In Houston DS conducted 83% of visit 2 DAREs with two other APPs conducting the remainder.

After conducting the DARE, the clinician provided DARE results and any needed referrals. The participant received $80 for their participation in the study. A total of 23 individuals completing the ASE and 3 couples completing the ACE reported not completing their exams at home when they arrived at the clinic. All 29 completed the exam at the beginning of the clinic visit before the clinician's DARE.

### Statistical methods

The frequencies of individuals’ characteristics completing both visit 1 and visit 2 ASE/ACE and who had valid DARE results at both visits were assessed. Loss to follow-up by participant characteristic was examined, as was the proportion in the practice arm who practiced at home between visit 1 and visit 2. In addition, we assessed the number of times the ACE/ACE was performed between visits.

Lay ASE/ACE examination results were compared with the clinician's DARE to derive the outcome of concordance.^7^ Participants were not included in the analysis if lay exam or clinician results were missing. For individuals, concordance was coded as “one” if the result of both clinician and participant was the same and “zero” if clinician and participant disagreed. For couples, concordance was coded as “one” if both clinician and the partner performing digital insertion agreed on the results for the other partner and “zero” if the performing partner and clinician disagreed. Cohen's kappa (*κ*) was calculated to determine agreement beyond chance between lay exam and DARE.^20^^,^^21^ Concordance and *κ* estimates with 95% confidence intervals (CI) were calculated for randomization arm, number of lay exams performed between visit 1 and visit 2, and by participant characteristics stratified by exam type, i.e., ASE or ACE. Differences in concordance by participant characteristics were assessed by chi-square tests of association, Fisher's exact test, or the Cochran–Armitage test for trend. All hypothesis tests were two-sided with a significance threshold p value of <0.05. We also assessed sensitivity and specificity of the lay exam.

In a sensitivity analysis to account for loss to follow-up, we used a multiple imputation approach^22^ to impute the clinician's DARE result and the participant's lay exam results for participants who did not return for visit 2. Similarly, we imputed data for covariates that showed a significant difference between participants retained on study and those lost to follow-up, i.e., age, city, clinician type, race/ethnicity, sexual orientation, and education (Supplementary Table S1). The imputed data were used to produce a κ estimate and 95% CI to compare with the overall κ estimate for those returning to visit 2.

The proportion of false negative, false positive, true positive, and true negative results for visit 1 and visit 2 was assessed with McNemar's test for dependent observations. The difference between visit 1 and visit 2 accuracy was assessed using the area under the receiver operating characteristic curve (AUC).

A priori power in the PAC Palpation study was assessed for sensitivity and specificity of the ASE and ACE at visit 1 rather than at visit 2^16^; thus, sample size was determined by the primary analysis. Analyses were conducted using SAS 9.4 TS Level 1 M6 (SAS Institute, Cary, NC), SPSS Version 29.0 (SPSS Inc., Chicago, Illinois), and R (Foundation for Statistical Computing, Vienna, Austria). The article followed STARD guidelines for research on diagnostic accuracy.^23^

### Ethics approval

Study procedures were approved by human protections committees at the Medical College of Wisconsin (PRO00033000), the University of Chicago, The University of Texas MD Anderson Cancer Center, and the University of Texas Health Sciences Center.

### Role of the funding source

The funder had no role in the study design, data collection, data analysis, interpretation, or writing of the manuscript.

## Results

Of 714 individuals randomized at baseline (Visit 1), 561 returned to complete visit 2, 79% (281/357) in the practice arm and 78% (280/357) in the control arm, respectively (Supplementary Figure S1). Compared to those retained on study, those lost to follow-up were younger (means of 42 years and 37 years, respectively, p = 0.001), and more likely to live in Chicago than Houston: 64% (98/153) and 36% (55/153), respectively, p < 0.001). Those retained in the study were less likely to be non-Hispanic Black (22%, 123/558) than those lost to follow-up (28%, 43/152, p = 0.01) and less likely to identify as bisexual (7%, 41/560 and 17%, 26/153, respectively, p = 0.001). Loss to follow-up was not associated with HIV status, presence of an abnormality at visit 1 or exam results at visit 1 (e.g., true negative or false positive). Loss to follow-up was also not associated with plans to do the ASE/ACE in the future (p = 0.11) or preference for a clinician's DARE rather than the lay exam (p = 0.14) (Supplementary Table S1).

In the practice arm, 89% (251/281) of participants reported practicing the ASE/ACE at home after three months (Table 1). Six of these reported a new abnormality not detected at visit 1. Five of these were seen in the study clinic with the clinician confirming an abnormality in 4. Two of these needed referrals for anal canal abnormalities. In the control arm 82% (229/278) of participants reported performing the ASE/ACE ≥ one time between visits.

**Table 1:** Characteristics of participants who completed visit 1 and visit 2, by randomization arm, in the Prevent Anal Cancer Palpation Study, Chicago, Illinois and Houston, Texas, USA, 2020–2023.

	Total (n = 561)	Practice (n = 281)	Control (n = 280)	p^a^
	n (col %)	n (col %)	n (col %)	
**Age**, years, median (IQR)	42 (33–54)	42 (33–54)	43 (32–55)	0.92^b^
**City**				0.64
Chicago	272/561 (49)	139/281 (50)	133/280 (48)	
Houston	289/561 (52)	142/281 (51)	147/280 (53)	
**Exam type**				0.35
Anal self-examination	515/561 (92)	261/281 (93)	254/280 (91)	
Anal companion exam	46/561 (8)	20/281 (7)	26/280 (9)	
**Race/ethnicity**				0.17
White, non-Hispanic	270/558 (48)	132/279 (47)	138/279 (50)	
Black, non-Hispanic	123/558 (22)	61/279 (22)	62/279 (22)	
Hispanic/Latino	125/558 (22)	71/279 (26)	54/279 (19)	
Asian, non-Hispanic	31/558 (6)	10/279 (4)	21/279 (8)	
Other, non-Hispanic^c^	9/558 (2)	5/279 (2)	4/279 (1)	
**Gender identity**				0.74^d^
Man (cis)	532/560 (95)	266/281 (95)	266/279 (95)	
Non-binary	12/560 (2)	8/281 (3)	4/279 (1)	
Transgender man or man^e^	7/560 (1)	3/281 (1)	4/279 (1)	
Transgender woman or woman^f^	8/560 (1)	4/281 (1)	4/279 (1)	
Other	1/560 (0)	0	1/279 (0)	
**Sexual orientation**				0.38^c^
Gay	487/560 (87)	243/281 (87)	244/279 (88)	
Bisexual	41/560 (7)	20/281 (7)	21/279 (8)	
Queer	25/560 (5)	16/281 (6)	9/279 (3)	
Other^g^	7/560 (1)	2/281 (1)	5/279 (2)	
**Education**, years				0.72
≤12	67/555 (12)	36/279 (13)	31/276 (11)	
13–15	116/555 (21)	60/279 (22)	56/276 (20)	
16	128/555 (23)	59/279 (21)	69/276 (25)	
>16	244/555 (44)	124/279 (44)	120/276 (44)	
**HIV status**				0.70
Negative	347/553 (63)	176/277 (64)	171/276 (62)	
Positive	206/553 (37)	101/277 (37)	105/276 (38)	
**Practiced at 3 months**				n/a
Yes	252/282 (89)	251/281 (89)	1/1 (100)^h^	
No	30/282 (11)	30/281 (11)	n/a	
**Performed ASE/ACE between visits, binary**				<0.001
Yes	495/557 (89)	266/279 (95)	229/278 (82)	
No	62/557 (11)	13/279 (5)	49/278 (18)	
**Performed ASE/ACE between visits, ordinal**				<0.001
0 times	62/557 (11)	13/279 (5)	49/278 (18)	
1 time	132/557 (24)	47/279 (17)	85/278 (31)	
≥2 times	363/557 (65)	219/279 (79)	144/278 (52)	
**Individuals with abnormalities at visit 2**				0.71
Yes	136/561 (24)	70/281 (25)	66/280 (24)	
Individuals with abnormalities getting a referral	20/561 (15)	11/281 (16)	9/280 (14)	
No	425/561 (76)	211/281 (75)	214/280 (76)	
**Visit 2 abnormality size**^i^ millimeters, median (IQR)	3 (2–4)	3 (2–4)	3 (2–4)	
**Visit 2 anal canal abnormality types** (n = 50)^j^				0.46
Hemorrhoid	28/50 (56)	15/28 (54)	13/22 (59)	
Scar	8/50 (16)	6/28 (21)	2/22 (9)	
Condyloma	5/50 (10)	4/28 (14)	1/22 (5)	
Suspicious thickening	3/50 (6)	1/28 (4)	2/22 (9)	
Papule	3/50 (6)	1/28 (4)	2/22 (9)	
Suspicious lump	1/50 (2)	1/28 (4)	0	
Skin tag	1/50 (2)	0	1/22 (5)	
Skin fold/flap	1/50 (2)	0	1/22 (5)	
**Visit 2 perianus abnormality types** (n = 97)^j^				0.89
Hemorrhoid	40/97 (41)	17/48 (35)	23/49 (47)	
Skin tag	34/97 (35)	17/48 (35)	17/49 (35)	
Condyloma	8/97 (8)	5/48 (10)	3/49 (6)	
Scar	7/97 (7)	3/48 (6)	4/49 (8)	
Papule	3/97 (3)	2/48 (4)	1/49 (2)	
Perianal ulcer	1/97 (1)	1/48 (2)	0	
Anal fissure	1/97 (1)	1/48 (2)	0	
Anal fistula	1/97 (1)	1/48 (2)	0	
Other	2/97 (2)	1/48 (2)	1/49 (2)	
**Visit 2 exam result**				0.96
True negative	415/561 (74)	206/281 (73)	209/280 (75)	
True positive	120/561 (21)	61/281 (22)	59/280 (21)	
False negative	16/561 (3)	9/281 (3)	7/280 (3)	
False positive	10/561 (2)	5/281 (2)	5/280 (2)	

a p value is derived from the chi-square test unless otherwise specified and does not include missing values.

b T-test of means.

c Other, non-Hispanic includes Native Hawaiian or other Pacific Islander, American Indian/Alaskan Native, and other.

d Fisher exact test.

e Individuals identifying as either a transgender man or as a man when sex assigned at birth is female.

f Individuals identifying as either a transgender woman or as a woman when sex assigned at birth is male.

g Other includes lesbian, heterosexual or straight, don't know, and other. Regardless of self-identified sexual orientation all participants met inclusion criteria.

h One participant in control arm was incorrectly asked to practice and they did.

i Size of lateral dimension.

j Unit of analysis is number of abnormality types. Since some individuals had abnormalities at both the anal canal and perianus, the summed number of abnormalities at each site is greater than the total number of individuals with an abnormality.

After stratifying participants returning for visit 2 by study arm, there were no notable differences in individual characteristics by arm (Table 1). For example, the proportion with an abnormality detected by DARE at visit 2 was 25% (70/281) and 24% (66/280) in the practice and control arms, respectively (p = 0.71). In addition, true negative, true positive, false negative, and false positive results did not differ by study arm (p = 0.96) (Table 1).

Overall concordance between clinician DARE and the participant ASE/ACE was 95% (535/561). Agreement between clinician DARE and participant ASE/ACE was almost perfect overall and in both study arms (*κ* = 0.87, 95% CI 0.82–0.92, overall; *κ* = 0.86, 95% CI 0.80–0.93, practice; and *κ* = 0.88, 95% CI 0.81–0.95, control); thus, there was no association between randomization arm and concordance (p = 0.69) (Table 2). Similarly, concordance was high for those doing the ASE in both study arms (95% (248/261) and 97% (245/254) in practice and control arms, respectively) and among those doing the ACE (95% (19/20) and 89% (23/25) in the practice and control arms, respectively) (Supplementary Table S2). Overall sensitivity was 0.88 (95% CI 0.83–0.94) and specificity was 0.98 (0.96–0.99).

**Table 2:** Concordance and agreement (κ) between clinician DARE result and lay exam result at visit 2 in the Prevent Anal Cancer Palpation Study, Chicago, Illinois and Houston, Texas, USA, 2020–2023.

	Concordant	Not concordant	κ (95% CI)	Sensitivity	Specificity
	n (row %)	n (row %)			
**Overall**	535/561 (95)	26/561 (5)	0.87 (0.82–0.92)	0.88 (0.83–0.94)	0.98 (0.96–0.99)
**Randomization arm**					
Practice	267/281 (95)	14/281 (5)	0.86 (0.80–0.93)	0.87 (0.79–0.95)	0.98 (0.96–0.99)
Control	268/298 (96)	12/298 (4)	0.88 (0.81–0.95)	0.89 (0.82–0.97)	0.98 (0.96–0.99)
	p = 0.69				
**Performed ASE/ACE between visits**					
0 times	55/62 (89)	7/62 (11)	0.73 (0.55–0.92)	0.75 (0.56–0.94)	0.95 (0.89–1.00)
1 time	123/132 (93)	9/132 (7)	0.78 (0.64–0.92)	0.81 (0.66–0.96)	0.96 (0.93–0.99)
≥2 times	354/363 (97)	9/363 (3)	0.93 (0.89–0.98)	0.93 (0.88–0.98)	0.99 (0.98–1.00)
	p < 0.001^a^				
**ASE/ACE location**					
Home	507/532 (95)	25/532 (5)	0.87 (0.82–0.92)	0.89 (0.68–1.00)	1.00
Clinic	28/29 (97)	1/29 (4)	0.92 (0.76–1.00)	0.88 (0.83–0.94)	0.98 (0.96–0.99)
	p = 0.99				
**Clinician type**					
Medical doctor	262/266 (99)	4/266 (2)	0.96 (0.92–1.0)	0.95 (0.89–1.00)	0.98 (0.97–1.00)
Advanced practice provider	272/295 (92)	23/295 (8)	0.79 (0.70–0.87)	0.84 (0.76–0.92)	0.97 (0.95–0.99)
	p < 0.001				
**Persistence, incidence, and clearance at visit 1 and visit 2**					
No abnormalities	337/340 (99)	3/340 (1)	n/a	n/a	n/a
Persistent abnormalities	93/109 (85)	16/109 (15)	n/a	n/a	n/a
Incident abnormalities	27/27 (100)	0	n/a	n/a	n/a
Clearing abnormalities	78/85 (92)	7/85 (8)	n/a	n/a	n/a
	p < 0.001^b^				
**City**					
Chicago	266/272 (98)	6/272 (2)	0.94 (0.89–0.99)	0.94 (0.88–0.99)	0.99 (0.98–1.00)
Houston	269/289 (93)	20/289 (7.0)	0.81 (0.73–0.89)	0.83 (0.74–0.92)	0.96 (0.94–0.99)
	p = 0.008				
**Age, years**					
25–34	177/184 (96)	7/184 (4)	0.85 (0.73–0.96)	0.82 (0.68–0.96)	0.99 (0.97–1.00)
35–44	117/121 (97)	4/121 (3)	0.92 (0.85–1.00)	0.97 (0.92–1.00)	0.96 (0.93–1.00)
45–54	116/124 (94)	8/124 (7)	0.84 (0.73–0.95)	0.86 (0.74–0.97)	0.97 (0.93–1.00)
≥55	125/132 (95)	7/132 (5)	0.87 (0.77–0.96)	0.86 (0.75–0.98)	0.98 (0.95–1.00)
	p = 0.34^b^				
**Race/ethnicity**					
White, non-Hispanic	255/270 (94)	15/270 (6)	0.85 (0.78–0.92)	0.88 (0.80–0.96)	0.97 (0.94–0.99)
Black, non-Hispanic	118/123 (96)	5/123 (4)	0.87 (0.76–0.98)	0.85 (0.71–0.98)	0.99 (0.97–1.00)
Hispanic/Latino	120/125 (96)	5/125 (4)	0.89 (0.80–0.98)	0.91 (0.81–1.00)	0.98 (0.95–1.00)
Asian, non-Hispanic	31/31 (100)	0	1.00	1.00	1.00
Other, non-Hispanic^c^	8/9 (89)	1/9 (11)	0.73 (0.24–1.00)	0.90 (0.71–1.00)	1.00
	p = 0.55				
**Gender identity**					
Man (cis)	508/532 (96)	24/532 (5)	0.88 (0.83–0.93)	0.88 (0.82–0.94)	0.98 (0.97–0.99)
Non-binary	12/12 (100)	0	1.00	1.00	1.00
Transgender man or man^d^	5/7 (71)	2/7 (29)	0.36 (−0.21–0.94)	1.00	0.67 (0.29–1.00)
Transgender woman or woman^e^	8/8 (100)	0	1.00	1.00	1.00
Other	1/1 (100)	0	1.00	1.00	1.00
	p = 0.13^a^				
**Sexual orientation**					
Gay	463/487 (95)	24/487 (5)	0.87 (0.82–0.92)	0.88 (0.83–0.94)	0.98 (0.96–0.99)
Bisexual	39/41 (95)	2/41 (5)	0.83 (0.60–1.00)	0.86 (0.60–1.00)	0.97 (0.91–1.00)
Queer	25/25 (100)	0	1.00	1.00	1.00
Other^f^	7/7 (100)	0	1.00	1.00	1.00
	p = 0.85^a^				
**Education, years**					
≤12	64/67 (96)	3/67 (5)	0.83 (0.65–1.00)	0.75 (0.51–0.99)	1.00
13–15	108/116 (93)	8/116 (7)	0.80 (0.67–0.93)	0.79 (0.63–0.94)	0.98 (0.95–1.00)
16	123/128 (96)	5/128 (4)	0.91 (0.84–0.99)	0.95 (0.89–1.00)	0.97 (0.93–1.00)
>16	234/244 (96)	10/244 (4)	0.88 (0.80–0.95)	0.90 (0.82–0.98)	0.97 (0.95–0.99)
	p = 0.58				
**HIV status**					
Negative	331/347 (95)	16/347 (5)	0.88 (0.80–0.95)	0.88 (0.81–0.95)	0.97 (0.96–0.99)
Positive	196/206 (95)	10/206 (5)	0.87 (0.80–0.93)	0.88 (0.79–0.96)	0.98 (0.96–1.00)
	p = 0.90				

p value is derived from the chi-square test unless otherwise specified.

a Cochran–Armitage Trend test.

b Fisher's exact test.

c Other, non-Hispanic includes Native Hawaiian or other Pacific Islander, American Indian/Alaskan Native, and other.

d Individuals identifying as either a transgender man or as a man when sex assigned at birth is female.

e Individuals identifying as either a transgender woman or as a woman when sex assigned at birth is male.

f Other includes lesbian, heterosexual or straight, don't know, and other. Regardless of self-identified sexual orientation all participants met inclusion criteria.

No serious adverse events were reported. In the sensitivity analyses, *κ* was 0.90 (95% CI 0.88–0.90), indicating that the study's loss to follow-up is unlikely to have substantially altered point estimates or conclusions.

**Fig. 1:**
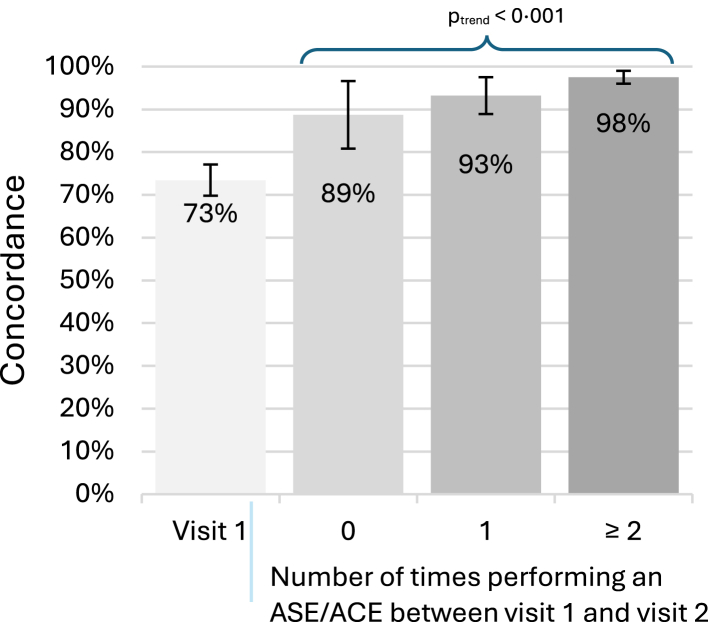
Concordance between anal self-exam or anal companion exam (ASE/ACE) and clinician digital anal rectal examination at visit 1 and by number of times an ASE/ACE was performed between visit 1 and 2 in the Prevent Anal Cancer Palpation Study, Chicago, Illinois and Houston, Texas, USA, 2020–2023.

Overall concordance increased from visit 1 to visit 2. There was a strong association between increased frequency of performing the ASE/ACE and concordance (p < 0.001) (Table 2 and Fig. 1).

Overall concordance for ASE/ACE was not associated with most other exposures; however, type of clinician was associated with concordance with medical doctors having higher concordance than advanced practice providers, 99% (262/266) and 92% (272/295), respectively (p < 0.001). Houston participants had lower concordance than those in Chicago (93% (269/289) and 98% (266/272), respectively, p = 0.008).

**Fig. 2:**
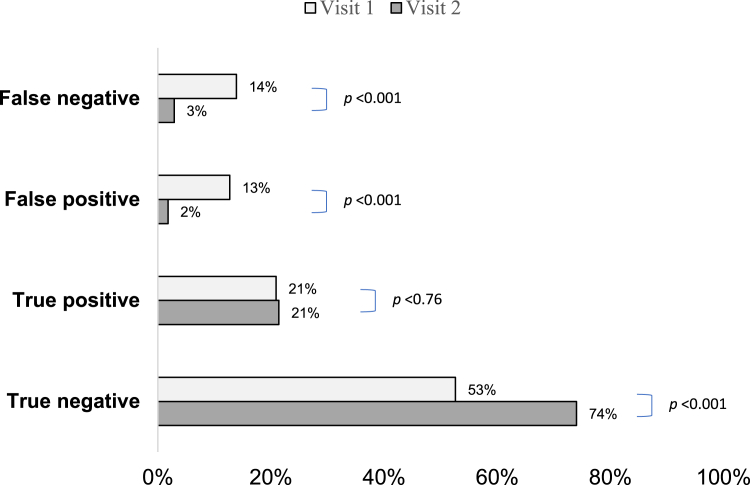
Change in anal self-exam and anal companion exam results from visit 1 to visit 2 in the Prevent Anal Cancer Palpation Study, Chicago, Illinois and Houston, Texas, USA, 2020–2023. p-value derived from McNemar's test.

The percentage of lay exams with false negative results declined from visit 1 to visit 2 (14% (78/561) to 3% (16/561), respectively, p < 0.001) in paired analysis for participants completing both visit 1 and visit 2 (Fig. 2). Likewise, false positive results for the ASE/ACE declined from 13% (71/561) to 2% (10/561), p < 0.001. The percentage of true negative ASE/ACE results increased from visit 1 to visit 2 (53% (295/561) to 74% (415/561), respectively, p < 0.001). Similar trends between visit 1 and visit 2 were observed for ASE and ACE separately (Supplementary Table S3).

**Fig. 3:**
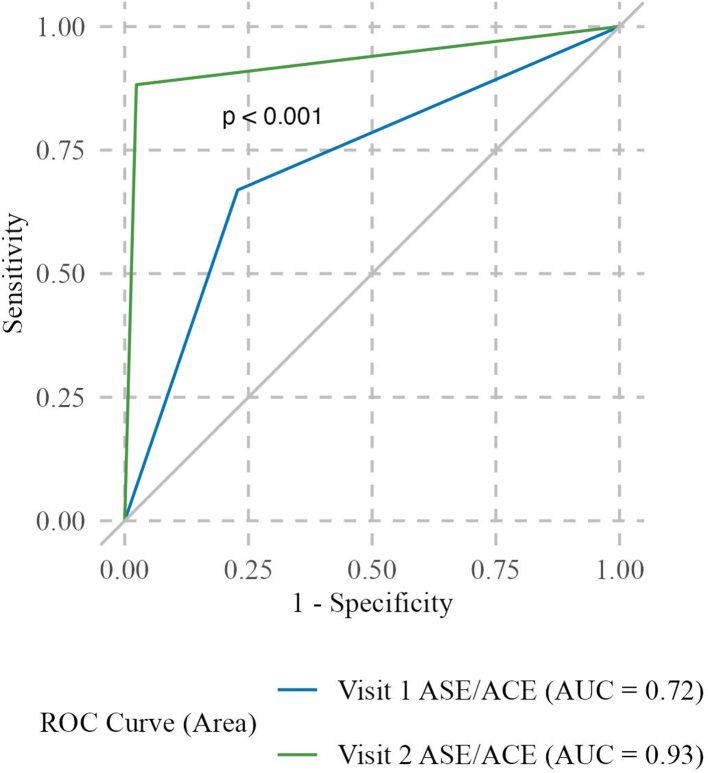
Area under the receiver operating characteristic curve (ROC) comparing anal self-exam and anal companion exam (ASE/ACE) results from visit 1 and visit 2 in the Prevent Anal Cancer Palpation Study, Chicago, Illinois and Houston, Texas, USA, 2020–2023.

The accuracy of participants conducting the ASE/ACE increased substantially from visit 1 to visit 2 (*κ*, 0.72 to 0.93, respectively, p < 0.001) (Fig. 3). Regarding preferred body position for conducting the ASE/ACE, the accuracy of the standing position (for example, standing in a shower or standing and putting one leg on the toilet) was highest (AUC 0.95, 95% CI 0.91–0.99) although the 95% CI for the AUC of other positions overlapped with the standing position (data not shown).

## Discussion

Lay anal exam and clinician anal exam results were concordant for 95% of participants with concordance significantly increasing from visit 1 to visit 2, regardless of randomization to practice or control arms. The proportions of false negatives and false positives were below 3%. There was a trend toward increasing concordance with the number of times an individual performed the lay exam. For those performing the ASE/ACE ≥2 times between visits, concordance was 98%. Overall agreement (κ) between lay exam and clinician exam was 0.87, almost perfect, and did not differ by age or HIV status. This implies that sexual minority men and transgender women may self-detect small lesions and thus downstage SCCA. Given the estimated sensitivity of 90% for DARE,^19^ we cannot exclude the possibility that the ASE/ACE is a superior mode for detecting palpable anal abnormalities compared with DARE.

Reasons for the increased concordance from visit 1 to visit 2 may include the visit 1 activities, familiarity with the exam, home-based location of the visit 2 exam, and recall of the visit 1 results. It is possible that visit 1 activities, including the clinician's explanation of the DARE results compared to the lay exam results, instilled confidence in the individuals doing the lay exam thereafter. This idea is supported by high concordance (89%) even among participants who reported not performing the ASE/ACE between visits. Also, when individuals performed the ASE/ACE at home, they would have been more familiar with the exam and possibly more comfortable doing the exam at home than in a clinic/office setting; however, we saw little difference in concordance between those who completed the exam at home as instructed versus the 29 individuals who needed to complete the exam in the clinic. Finally, some individuals may have remembered their visit 1 results which may have influenced their visit 2 result six months later; however, among 27 participants with an incident abnormality, i.e., the clinician detected an abnormality at visit 2 but not visit 1, all 27 successfully detected the incident abnormality. Also, the clinician's DARE indicated that a total of 85 individuals had an abnormality at visit 1 that cleared by visit 2. Most of these (87%) were skin folds, skin flaps, skin tags, or haemorrhoids. Of the 85 individuals, 78 (92%) correctly reported after their lay exam that the abnormality had cleared (Table 2).

The percentage of abnormalities detected by clinicians declined from visit 1 (34%^16^) to visit 2 (24%) although there was no association between presence of an abnormality at visit 1 and retention at visit 2. Likewise, there was no association between lay exam result at visit 1, for example, true negative or false positive, and retention at visit 2. We believe the proportion of abnormalities largely declined due to clearance of 85 abnormalities detected at visit 1 coupled with 27 incident abnormalities (Table 2).

The lesions successfully palpated by individuals were a median of 3 mm in size as in visit 1,^16^ indicating that even superficially invasive squamous cell carcinomas of the anus may be detected by lay self-palpation as they are by clinician palpation.^24^

Recognition of symptoms may increase anal precancer screening uptake, while unnecessary clinical visits, for example, for time-limited haemorrhoids, may be minimal after ASE/ACE: 74% of participants said that after detecting an abnormality, they would wait up to a week to see if the problem resolved before making a doctor's appointment (data not shown).

Study arm, i.e., practice or control, was not associated with concordance, contrary to the hypothesis, possibly because 82% of individuals in the control arm also reported performing the ASE/ACE between visits, although they were not encouraged to do so. Both city and clinician type were significantly associated with concordance. These variables were highly correlated since most DAREs in Chicago were conducted by a medical doctor while all DAREs in Houston were conducted by an advanced practice provider (80% and 100%, respectively, *r*_spearman_ = 0.82). Clinician type was not associated with the practice of ASE/ACE between visits, or the number of times ASE/ACE was performed between visits. Medical doctors may have had higher concordance than APPs because of increased medical training.

We believe the current results, in addition to our prior studies,^16^^,^^17^ indicate that sexual minority men are highly accomplished at recognizing abnormalities at the perianus or in the anal canal; thus, we believe that the ASE/ACE is a self-care behaviour that clinicians should suggest to patients at increased risk for SCCA for a number of reasons: Concordance with a clinician's exam is high, individuals can detect abnormalities much smaller than 10 mm when treatment is much easier for SCCA,^25^^,^^26^ anxiety and pain is minimal among those conducting the exams,^27^ acceptability is high,^28^ and participants may have a preference for being taught the procedure by a clinician.^17^^,^^27^ The lay examinations may also be useful after treatment of precancers or SCCA to support early recognition of invasive disease or recurrence. It may be especially helpful in regions with high HIV prevalence or SCCA incidence yet inadequate infrastructure for anal precancer screening, for example, the US Midwest, South, and sub-Saharan Africa. We suggest caution in interpreting these results for non-cisgender individuals since only 5% of our sample was gender diverse.

While it is unlikely that clinics will have time to devote to anal cancer education and training of patients, the clinician can use the DARE as a teachable moment, e.g., “a person's anal canal is relatively short so I'm only inserting my finger to the second knuckle to reach the full length of the anal canal. I'm making sure to feel 360° around the anal canal for anything that's not smooth, like a hard spot or a lump, even as small as a grain of rice.” The above wording reflects instructions to the lay person used in the study's training (Supplementary Figures S2 and S3). Almost one-half (46%) of the current sample at visit 1 reported anal self-palpation for disease before joining the study, indicating a high proportion of individuals are already conducting an anal examination, presumably without benefit of training or anal pathology education.^16^ Anal cancer education is needed for these individuals given reports of substantial anxiety after getting abnormal anal cancer screening results in clinics.^29^

While we observed higher concordance at visit 1 when clinicians conducted the ASE/ACE training compared to non-clinician staff,^16^ trainer type was not associated with concordance at visit 2, suggesting that non-clinician training may be sufficient. Also, unlike visit 1, where we observed a decline in concordance of the ASE/ACE with increasing age,^16^ no such association was seen at visit 2. It is possible that the association disappeared because of the clinician's consult about the individual's ASE/ACE result at the end of visit 1, or because of the additional comfort of doing the ASE/ACE at home. Currently recommended ages to begin anal precancer screening are at 35 years for sexual minority men and transgender women with HIV and 45 years for those without HIV. Note that among populations for whom SCCA is very rare, e.g., people with HIV aged <35 years, non-malignant and common palpable and visible abnormalities such as condyloma may also be detected by ASE/ACE.^16^

ASE/ACE cannot be further assessed using a randomized clinical trial with outcomes of SCCA due to ethical considerations and cost; however, recent research from the ANCHOR^30^ study indicates a significant association between an abnormality detected by DARE and large precancers that are most likely to invade^31^; thus, trials of the utility of ASE/ACE with an outcome of precancer are warranted. In addition, future research needs to assess the implementation aspects of the ASE/ACE and the potential to teach the ASE/ACE using online tutorials. Finally, ASE/ACE might be used with other populations recommended for screening, although data on acceptability is sparse outside of sexual minority men and transgender populations.^32^

### Limitations

Limitations to the study include retention, which was 79%. We did not know the reasons for not attending visit 2, although we contacted individuals three times to reschedule and/or learn the reasons for non-attendance. Although retention was associated with some individual characteristics like age and race, it was not associated with visit 1 abnormalities or ASE/ACE results. Another limitation is that the convenience sampling for this study may have attracted individuals who were more interested in prevention of anal cancer. Finally, the DARE results are dependent upon the performer, which is possibly illustrated in this study by the physician having higher concordance with lay exams than APPs; although, concordance was over 90% for both.

In conclusion, six months after learning how to conduct the ASE/ACE, individuals at highest risk for SCCA had very high agreement between their lay exam and a DARE conducted by a clinician. False negative and false positive results for the ASE/ACE were below 3% each. Concordance was not associated with study arm but increasing concordance was associated with the number of times performing the ASE/ACE between visits. We recommend that when clinicians conduct DARE, they use it as a teachable moment to suggest to patients at increased risk for SCCA that their own lay exams may be useful in detecting intra-anal and perianal lesions.

## Contributors

AGN conceptualized the study, secured funding, and had final responsibility for the decision to submit the manuscript for publication. AGN, MDS, TLM, and EYC developed the methodology. CL, AH, and JK administered the clinics and taught participants how to do the lay examinations. AH and DS conducted clinical examinations. AGN, and CL managed the data. AGN, DY, JN, and TLM had access to the data and conducted the analysis. AGN, DY, and JN verified the data. AGN wrote the first draft of the manuscript. AGN, TLM, JN, CL, MDS, AAD, EYC, JAS, JK, EA, JMW, LH, DS, and AH reviewed and edited manuscript drafts.

## Data sharing statement

Fully de-identified datasets, data dictionary, and protocol will be shared with properly trained investigators on the study website (https://mindyourbehind.org) within 1 year of study completion after assessment of institutional policies, Medical College of Wisconsin Human Research Protections Program rules, as well as local, state, and federal laws and regulations. Further information is available from the corresponding author upon request.

## Declaration of interests

AGN declared receiving consulting fees from Merck & Co. for purposes not related to this article in addition to support from EUROGIN for conference registration fees and lodging; AH declared receiving consulting fees from Gilead Sciences, ViiV Healthcare, and Abbott Technologies; AAD declared receiving consulting fees from Merck Inc., Value Analytics Lab, support to attend EUROGIN, and payment or honoraria for giving talks/conferences at the NIH and Mt. Sinai; EYC has a leadership or fiduciary role as Chair of the Solid Tumor Working Group of the AIDS Malignancy Consortium.

## Appendix A Supplementary data

Supplementary Figures ad Tables

Protocol
