# Supplementary material for: The longitudinal effect of repetition and practice on the accuracy of lay anal examinations for detecting perianal and anal canal abnormalities: a prospective study
Source: Lancet Reg Health Am. 2025 Dec 6;53:101317. doi: 10.1016/j.lana.2025.101317 (PMC12732307; doi:10.1016/j.lana.2025.101317)

Supplementary Material

The longitudinal effect of repetition and practice on the accuracy of lay anal examinations for detecting perianal and anal canal abnormalities: A prospective study

Alan G. Nyitray, PhD; Timothy L. McAuliffe, PhD; Jenna Nitkowski, PhD; Cameron Liebert, APSW; Michael D. Swartz, PhD; Ashish A. Deshmukh, PhD; Jared Kerman; Ellen Almirol, MPH; John A. Schneider, MD; J. Michael Wilkerson, PhD; Lu-Yu Hwang, MD; Derek Smith, APRN; Duo Yu, PhD; Aniruddha Hazra, MD; Elizabeth Y. Chiao, MD for The Prevent Anal Cancer Palpation Study Team.

Contents

Supplementary Figure 1: Study flow

Supplementary Table 1: Characteristics of participants retained on study compared with those lost to follow-up in the Prevent Anal Cancer Palpation Study, Chicago, Illinois and Houston, Texas, USA, 2020-2023.

Supplementary Table 2: Concordance between clinician DARE result and lay exam result by lay exam type at visit 2 in the Prevent Anal Cancer Palpation Study, Chicago, Illinois and Houston, Texas, USA, 2020-2023.

Supplementary Table 3: Change in anal self-exam and anal companion exam results from paired visit 1 and visit 2 results and stratified by exam type in the Prevent Anal Cancer Palpation Study, Chicago, Illinois and Houston, Texas, USA, 2020-2023.

Supplementary Figure 2: Anal self-exam study instructions

Supplementary Figure 3: Anal companion exam study instructions

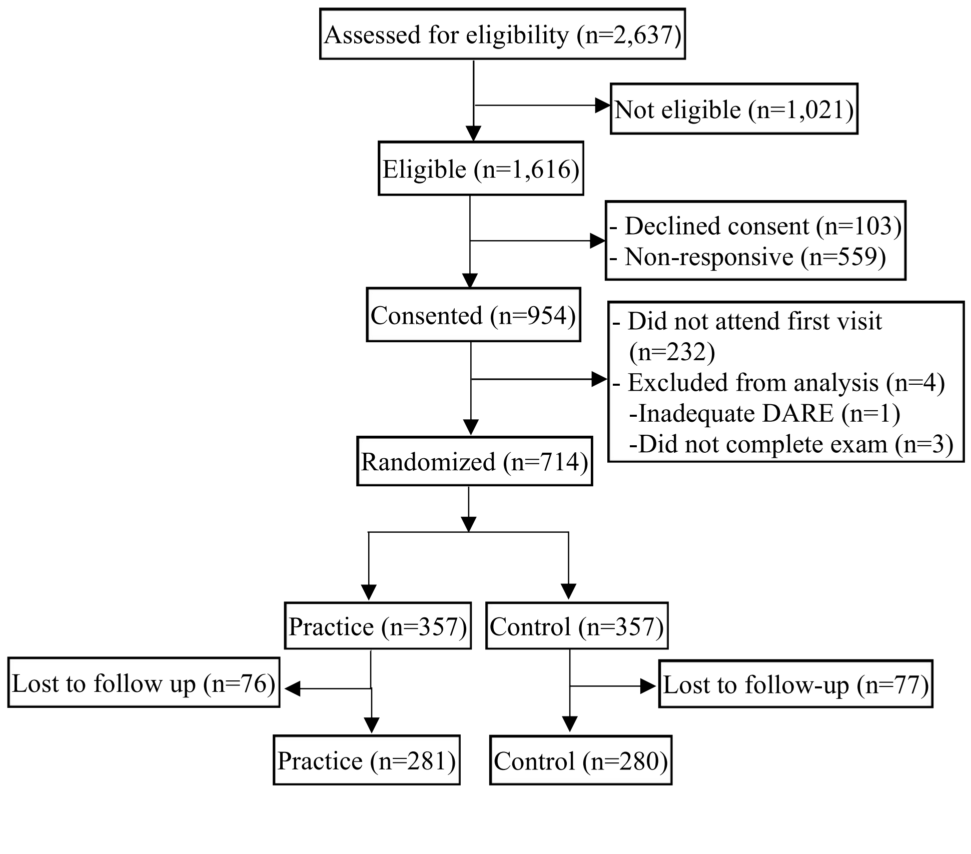

Supplementary Figure 1. Study flow.

Supplementary Table 1: Characteristics of participants retained on study compared with those lost to follow-up in the Prevent Anal Cancer Palpation Study, Chicago, Illinois and Houston, Texas, USA, 2020-2023.			
	**Retained n=561**	**Lost to follow-up n=153**	**p[a]**
	**n (col %)**	**n (col %)**	
**Age**, years, median (IQR)	42 (33-54)	37 (31-50)	0·001[b]
**City**			< 0·001
Chicago	272/561 (48)	98/153 (64)	
Houston	289/561 (52)	55/153 (36)	
**Exam type**			0·50
Anal self-examination	515/561 (92)	143/153 (94)	
Anal companion exam	46/561 (8)	10/153 (7)	
**Clinician type**			< 0·001
Medical doctor	218/561 (39)	83/153 (54)	
Advanced practice provider	343/561 (61)	70/153 (46)	
**Race/ethnicity**			0·01
White, non-Hispanic	270/558 (48)	64/152 (42)	
Black, non-Hispanic	123/558 (22)	43/152 (28)	
Hispanic/Latino	125/558 (22)	38/152 (25)	
Asian, non-Hispanic	31/558 (6)	1/152 (1)	
Other, non-Hispanic[c]	9/558 (2)	6/152 (4)	
**Gender identity**			0·13[d]
Man (cis)	532/560 (95)	139/153 (91)	
Non-binary	12/560 (2)	6/153 (4)	
Transgender man or man[e]	7/560 (1)	2/153 (1)	
Transgender woman or woman[f]	8/560 (1)	4/153 (3)	
Other	1/560 (0)	2/153 (1)	
**Sexual orientation**			0·001
Gay	487/560 (87)	114/153 (75)	
Bisexual	41/560 (7)	26/153 (17)	
Queer	25/560 (5)	10/153 (7)	
Other[g]	7/560 (1)	3/153 (2)	
**Education**, years			0·07
≤ 12	67/555 (12)	26/148 (18)	
13-15	116/555 (21)	40/148 (27)	
16	128/555 (23)	29/148 (20)	
>16	244/555 (44)	53/148 (36)	
**HIV status**			0·67
Negative	347/553 (63)	97/150 (65)	
Positive	206/553 (37)	53/150 (35)	
**DARE result at visit 1**			0·77
Normal	367/561 (65)	102/153 (67)	
Abnormal	194/561 (35)	51/153 (33)	
**ASE/ACE exam result at visit 1**			0·98
True negative	295/561 (53)	81/153 (53)	
True positive	117/561 (21)	30/153 (20)	
False negative	78/561 (14)	21/153 (14)	
False positive	71/561 (13)	21/153 (14)	
**Preference for ASE/ACE or doctor-provided exam**			0·14
Doctor-provided exam	356/556 (64)	105/149 (71)	
ASE or ACE	200/556 (36)	44/149 (20)	
**Plans to do ASE/ACE in the future**			0·11[d]
Strongly agree	405/559 (73)	103/150 (69)	
Agree	144/559 (26)	40/150 (27)	
Disagree	1/559 (0)	2/150 (1)	
Strongly disagree	2/559 (0)	0	
I don't know	7/559 (1)	5/150 (3)	
**Pain with the ASE/ACE**			0·27[d]
None	539/560 (96)	142/150 (95)	
A little	15/560 (3)	8/150 (5)	
A lot	2/560 (0)	0	
I don't know	4/560 (1)	0	
[a] Chi square unless otherwise specified·			
[b] T-test of means·			

[c] Other, non-Hispanic includes Native Hawaiian or other Pacific Islander, American Indian/Alaskan Native, and other.
[d] Fisher's exact test
[e] Individuals identifying as either a transgender man or as a man when sex assigned at birth is female.
[f] Individuals identifying as either a transgender woman or as a woman when sex assigned at birth is male.
[g] Other includes lesbian, heterosexual or straight, don't know, and other. Regardless of self-identified sexual orientation all participants met inclusion criteria.

Supplementary Table 2: Concordance between clinician DARE result and lay exam result by lay exam type at visit 2 in the Prevent Anal Cancer Palpation Study, Chicago, Illinois and Houston, Texas, USA, 2020-2023.				
	**Anal self-exam**		**Anal companion exam**	
	**Concordant**	**Not concordant**	**Concordant**	**Not concordant**
	**n (row %)**	**n (row %)**	**n (row %)**	**n (row %)**
**Overall**	493/515 (96)	22/515 (4)	42/46 (91)	4/46 (9)
**Randomization arm**				
Practice	248/261 (95)	13/261 (5)	19/20 (95)	1/20 (5)
Control	245/254 (97)	9/254 (4)	23/25 (89)	3/25 (12)
	p = 0·42		p = 0·62[a]	
**Performed ASE/ACE between visits**				
0 times	50/56 (89)	6/56 (11)	5/6 (83)	1/6 (17)
1 time	106/113 (94)	7/113 (6)	17/19 (90)	2/19 (11)
≥ 2 times	335/344 (97)	9/344 (3)	19/19 (100)	0
	p = 0·003[b]		p = 0·10[b]	
**ASE/ACE location**				
Home	471/492 (96)	21/492 (4)	36/40 (90)	4/40 (10)
Clinic	22/23 (96)	1/23 (4)	6/6 (100)	0
	p = 1·00[a]		p = 1·00[a]	
**Clinician type**				
Medical doctor	250/254 (98)	4/254 (2)	12/12 (100)	0
Advanced practice provider	242/261 (93)	19/261 (7)	30/34 (88)	4/34 (12)
	p = 0·002		p = 1·00[a]	
**Persistence, incidence, and clearance at visit 1 and visit 2**				
No abnormalities	313/316 (99)	3/316 (1)	24/24 (100)	0
Persistent abnormalities	85/101 (84)	16/101 (16)	8/8 (100)	0
Incident abnormalities	22/22 (100)	0	5/5 (100)	0
Clearing abnormalities	73/76 (96)	3/76 (4)	5/5 (56)	4 (44)
	p < 0·001[a]		p = 0·002[a]	
**City**				
Chicago	254/260 (98)	6/260 (2)	12/12 (100)	0
Houston	239/255 (94)	16/255 (6)	30/34 (88)	4/34 (12)
	p = 0·03		p = 0·56[a]	
**Age, years**				
25-34	164/171 (96)	7/171 (4)	13/13 (100)	0
35-44	104/106 (98)	2/106 (2)	13/15 (87)	2/15 (13)
45-54	109/116 (94)	7/116 (6)	7/8 (88)	1/8 (13)
≥ 55	116/122 (95)	6/122 (5)	9/10 (90)	1/10 (10)
	p = 0·46[b]		p = 0·42[b]	
**Race/ethnicity**				
White, non-Hispanic	227/239 (95)	12/239 (5)	28/31 (90)	3/31 (10)
Black, non-Hispanic	115/120 (96)	5/120 (4)	3/3 (100)	0
Hispanic/Latino	114/118 (97)	4/118 (3)	6/7 (86)	1/7 (14)
Asian, non-Hispanic	28/28 (100)	0	3/3 (100)	0
Other, non-Hispanic[c]	7/8 (88)	1/8 (13)	1/1 (100)	0
	p = 0·54		p = 1·00[a]	
**Gender identity**				
Man (cis)	469/489 (96)	20/489 (4)	39/43 (91)	4/43 (9)
Non-binary	12/12 (100)	0	0	0
Transgender man or man[d]	4/6 (67)	2/6 (33)	1/1 (100)	0
Transgender woman or woman[e]	7/7 (100)	0	1/1 (100)	0
Other	1/1 (100)	0	0	0
	p = 0·08[a]		p = 1·00[a]	
**Sexual orientation**				
Gay	425/445 (96)	20/445 (5)	38/42 (91)	4/42 (10)
Bisexual	36/38 (95)	2/38 (5)	3/3 (100)	0
Queer	24/24 (100)	0	1/1 (100)	0
Other[f]	7/7 (100)	0	0	0
	p = 0·75[a]		p = 1·00[a]	
**Education, years**				
≤ 12	59/62 (95)	3/62 (5)	5/5 (100)	0
13-15	102/109 (94)	7/109 (6)	6/7 (86)	1/7 (14)
16	113/116 (97)	3/116 (3)	10/12 (83)	2/12 (17)

>16	213/222 (96)	9/222 (4)	21/22 (96)	1/22 (5)
	p = 0·52		p = 1·00	
**HIV status**				
Negative	303/317 (96)	14/317 (4)	28/30 (93)	2/30 (7)
Positive	182/190 (96)	8/190 (4)	14/16 (88)	2/16 (13)
	p = 0·91		p = 0·43[a]	

p value is derived from the chi-square test unless otherwise specified.

[a] Fisher's exact test

[b] Cochran-Armitage Trend test

[c] Other, non-Hispanic includes Native Hawaiian or other Pacific Islander, American Indian/Alaskan Native, and other.

[d] Individuals identifying as either a transgender man or as a man when sex assigned at birth is female.

[e] Individuals identifying as either a transgender woman or as a woman when sex assigned at birth is male.

[f] Other includes lesbian, heterosexual or straight, don't know, and other. Regardless of self-identified sexual orientation all participants met inclusion criteria.

**Supplementary Table 3: Change in anal self-exam and anal companion exam results from paired visit 1 and visit 2 results and stratified by exam type in the Prevent Anal Cancer Palpation Study, Chicago, Illinois and Houston, Texas, USA, 2020-2023.**			
	**Visit 1**	**Visit 2**	
	**n (%)**	**n (%)**	p value[a]
**Anal self-exam**	n = 515	n = 515	
True negative	272/515 (53)	386/515 (75)	< 0·001
True positive	106/515 (21)	107/515 (21)	0·91
False negative	71/515 (14)	16/515 (3)	< 0·001
False positive	66/515 (13)	6/515 (1)	< 0·001
**Anal companion exam**	n = 46	n = 46	
True negative	23/46 (50)	29/46 (63)	0·08
True positive	11/46 (24)	13/46 (28)	0·53
False negative	7/46 (15)	0	-
False positive	5/46 (11)	4/46 (9)	0·74

[a] McNemar's test

Supplementary Figure 2: Anal self-exam study instructions

Anal Self-Exams

Step 1
The first thing is to try to look at your anus. Look for warts, skin tags, or a sore. You could look at your anus a number of ways:
- Use a smart phone in selfie or mirror mode to see your anus.
- Lean a mirror against a wall and then sit on the floor with your legs spread.
- Put a mirror on the floor between your legs as you spread your cheeks. Use a flashlight if you want.

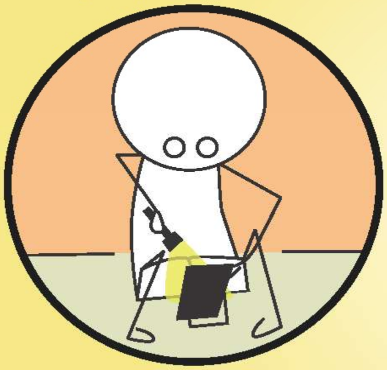

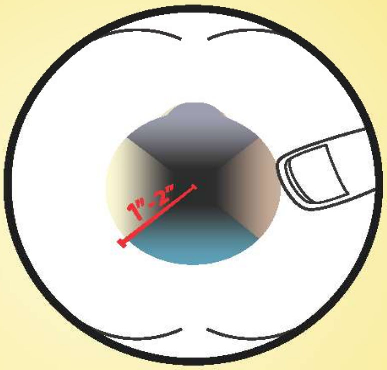

Step 2
Imagine that your anal canal is a cylinder and has four sections. You will use your finger to feel each section.

Step 3
Correctly put a glove on your dominant hand and put lube on your "pointing" or index finger.

Step 4 Positioning
There are several positions that may help you perform the examination effectively. Choose the position that is most comfortable and gives you the best access to your anus.

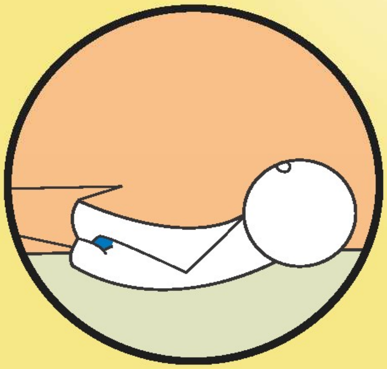

Position 1
Lay on your side. If you're right handed, lay on your left side and pull up your right leg. If you're left-handed, lay on your right side and pull up your left leg.

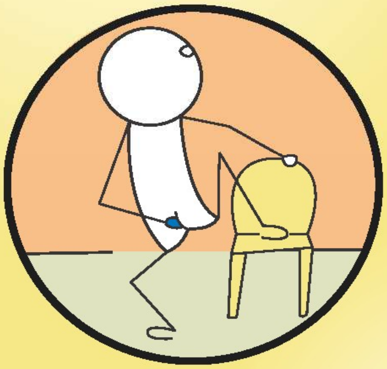

Position 2
Stand with one foot on a chair. If you're right-handed, put your right foot on the chair. If you're left-handed, put your left foot on the chair.

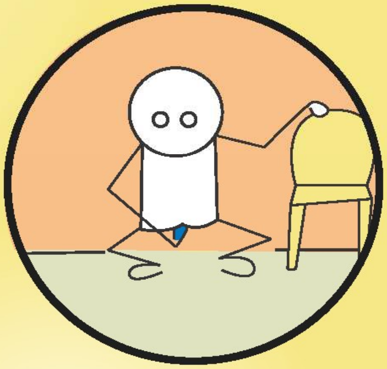

Position 3
Do the squat!

Step 5
Whichever position you choose, reach to press the pad of your index finger against the opening of your anus for a second or two. This helps your anus relax.

Step 6
Insert your index finger to the first knuckle. Feel as far around your anal canal as you can. Then insert your finger to the second knuckle and feel as far around as you can (360°).

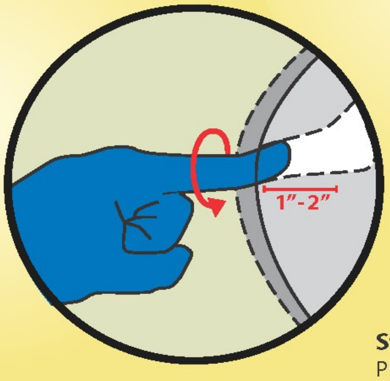

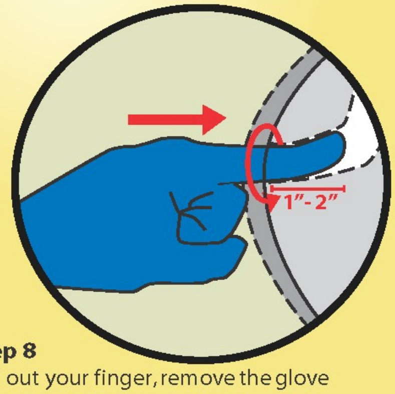

Step 7
Change hands and feel around the rest of your anal canal. (Lather, rinse, repeat!) It's important to feel 360° around your anal canal.

Step 8
Pull out your finger, remove the glove (correctly), wipe anus with gauze or tissue, and wash your hands.

Supplementary Figure 3: Anal companion exam study instructions

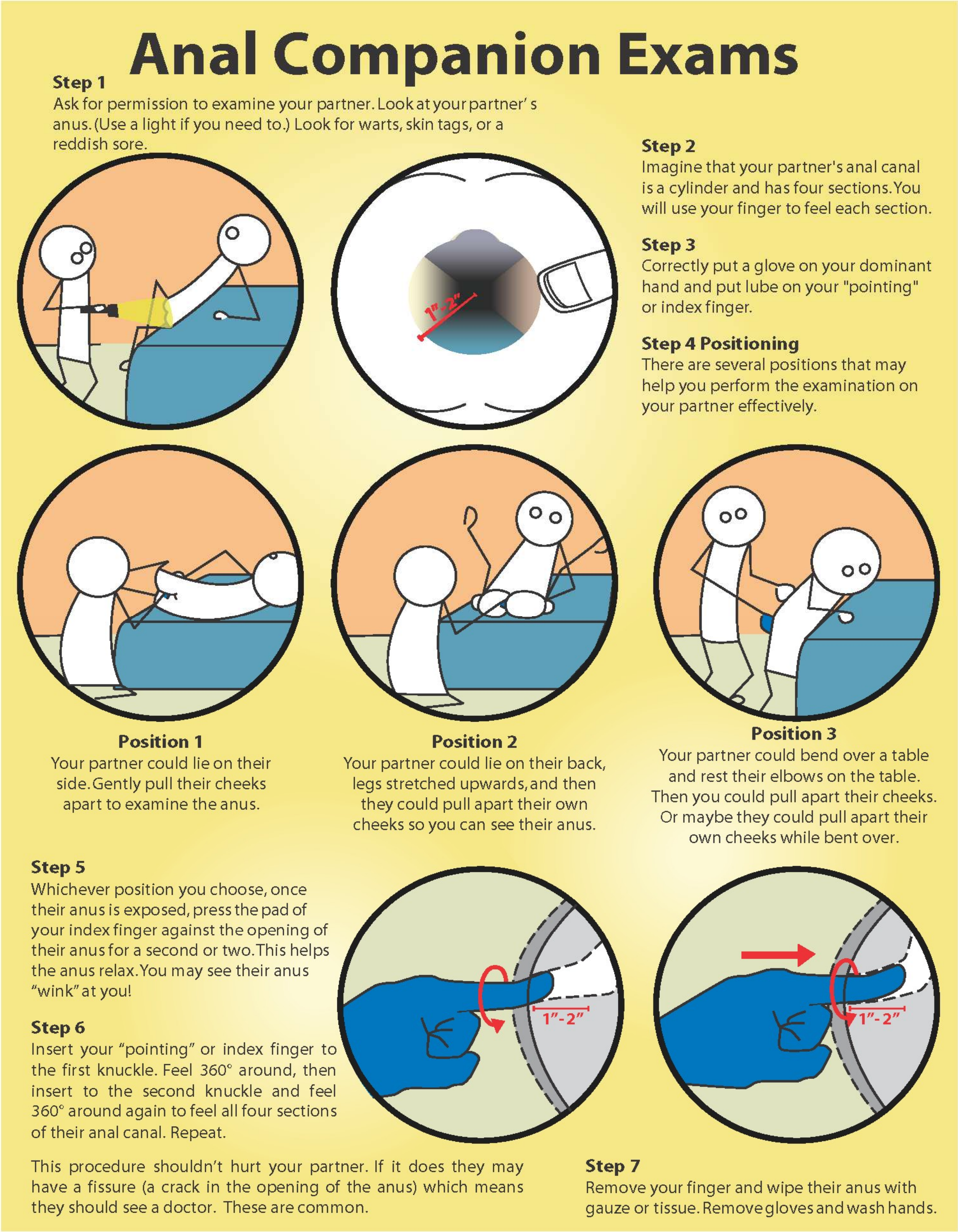

Supplement: Supplementary Figures ad Tables [file mmc1.pdf]
