# Supplementary material for: The longitudinal effect of repetition and practice on the accuracy of lay anal examinations for detecting perianal and anal canal abnormalities: a prospective study
Source: Lancet Reg Health Am. 2025 Dec 6;53:101317. doi: 10.1016/j.lana.2025.101317 (PMC12732307; doi:10.1016/j.lana.2025.101317)
Supplement: Protocol [file mmc2.docx]

**PROTOCOL**

**THE PREVENT ANAL CANCER (PAC) PALPATION STUDY**

**Sponsor:** National Cancer Institute of the National Institutes of Health

**Official title:** Determining the accuracy of self- and partner anal exams for detecting anal abnormalities.

**Grant number:** 1R01CA232892-01

**NCT Registration Number:** NCT04090060

**Principal Investigator:**

*Alan G. Nyitray, Ph.D.**Clinical Cancer Center, Center for AIDS Intervention Research*

*Medical College of Wisconsin*

*8701 Watertown Plank Road, Ste. 5400*

*Milwaukee, WI, 53226*

*Tel: 414-805-3312
Email: anyitray@mcw.edu*

**Co-Investigators:**

1. **Elizabeth Chiao, MD, MPH**

*Professor*

*The University of Texas MD Anderson Cancer Center*

*Houston, TX*

*Tel: 713-792-1860*

*E-Mail: eychiao@mdanderson.org*

1. **John Schneider, MD, MPH**

*Associate Professor*

*Departments of Medicine and Public Health Sciences*

*University of Chicago*

*5841 S. Maryland, MC 5065*

*Chicago, IL 60637*

*Tel: 1-773-702-8349*

*E-Mail: jschnei1@medicine.bsd.uchicago.edu*

1. **Aniruddha Hazra, MD**

*Assistant Professor of Medicine*

*Section of Infectious Diseases and Global Health*

*University of Chicago*

*5841 S. Maryland, MC 5065*

*Chicago, IL 60637*

*Tel:1-773-795-2016*

*E-mail: ahazra2@medicine.bsd.uchicago.edu*

1. **Ashish Deshmukh, PhD, MPH**

*Assistant* *Professor*

*The University of Texas Health Science Center at Houston School of Public Health*

*Department of Management, Policy, and Community Health*

*1200 Pressler Street, E-329*

*Houston, TX 77030*

*Tel:1-713-500-9180*

*E-mail: ashish.a.deshmukh@uth.tmc.edu*

1. **Michael Swartz, PhD**

*Associate Professor*

*The University of Texas Health Science Center at Houston School of Public Health*

*Department of Biostatistics and Data Science*

*1200 Pressler Street, E-815*

*Houston, TX 77030*

*Tel: 1-713-500-9570*

*Fax: 1-713-500-9525*

*E-mail: Michael.D.Swartz@uth.tmc.edu*

1. **Michael Wilkerson, PhD**

*Assistant Professor*

*The University of Texas Health Science Center at Houston School of Public Health*

*Department of* *Health Promotion and Behavioral Sciences*

*7000 Fannin Street, Rm 2620*

*Houston, TX 77030*

*Tel: 1-713-500-9974*

*Fax: 1-713-500-9602*

*E-mail: Johnny.M.Wilkerson@uth.tmc.edu*

1. **Lu-Yu Hwang, MD**

*Professor*

*The University of Texas Health Science Center at Houston School of Public Health*

*Department of Epidemiology, Human Genetics, and Environmental Sciences*

*1200 Pressler Street, E-717*

*Tel: 1-713-500-9382*

*E-mail: lu-yu.hwang@uth.tmc.edu*

Contents

[PROTOCOL CHANGE HISTORY 7](#_Toc58926006)

[LIST OF ABBREVIATIONS 7](#_Toc58926007)

[SITES PARTICIPATING IN THE STUDY 8](#_Toc58926008)

[PROTOCOL ROSTER 8](#_Toc58926009)

[Principal Investigator 8](#_Toc58926010)

[Co-Investigators 8](#_Toc58926011)

[STUDY SYNOPSIS 10](#_Toc58926012)

[MULTI-CENTER STUDY 10](#_Toc58926013)

[BACKGROUND 10](#_Toc58926014)

[STUDY PURPOSE 11](#_Toc58926015)

[Aim 1 11](#_Toc58926016)

[Aim 2 11](#_Toc58926017)

[Aim 3 11](#_Toc58926018)

[Exploratory aim 12](#_Toc58926019)

[OUTCOMES 12](#_Toc58926020)

[SAMPLE SIZE 12](#_Toc58926021)

[POPULATION 12](#_Toc58926022)

[DURATION OF PARTICIPANT ACTIVITIES 12](#_Toc58926023)

[PARTICIPANT ENROLLMENT 14](#_Toc58926024)

[Inclusion Criteria 14](#_Toc58926025)

[Exclusion Criteria 14](#_Toc58926026)

[Recruitment Procedures and Setting 14](#_Toc58926027)

[Referral Program 14](#_Toc58926028)

[Community Advisory Board 15](#_Toc58926029)

[Eligibility Procedures 16](#_Toc58926030)

[Visit 1 Overview 16](#_Toc58926031)

[Visit 1 Detail 17](#_Toc58926032)

[Randomization 19](#_Toc58926033)

[Arm 1 - Practice Arm 19](#_Toc58926034)

[Arm 2 – Non-practice Arm 20](#_Toc58926035)

[Between Visit 1 and Visit 2 study procedures 20](#_Toc58926036)

[Preparation for Visit 2 21](#_Toc58926037)

[Visit 2 Study Procedures 21](#_Toc58926038)

[Data Collected from Study Participants 22](#_Toc58926039)

[BURDEN OF TIME ON PARTICIPANTS DOING THE ASE 23](#_Toc58926040)

[STATISTICAL ANALYSIS 25](#_Toc58926041)

[Aim 1 25](#_Toc58926042)

[Aim 2 26](#_Toc58926043)

[Aim 3 26](#_Toc58926044)

[**PROTOCOL FIDELITY AND QUALITY CONTROL** 28](#_Toc58926045)

[HUMAN PROTECTIONS 29](#_Toc58926046)

[Overview 29](#_Toc58926047)

[Informed Consent Process 30](#_Toc58926048)

[Use and Access of Protected Health Information (PHI) and Retention of Records 30](#_Toc58926049)

[Risks to the Subjects 31](#_Toc58926050)

[Alternatives to study procedures 32](#_Toc58926051)

[Protections against risk 32](#_Toc58926052)

[Benefits 33](#_Toc58926053)

[Adverse Event Reporting 33](#_Toc58926054)

[Data and Safety Monitoring Committee 34](#_Toc58926055)

[**REPORTING TO COMMUNITIES** 35](#_Toc58926056)

#

| PROTOCOL CHANGE HISTORY | | | | |
| --- | --- | --- | --- | --- |
| **Date submitted** | **Date approved** | **Version** | **Revisions** | **Author** |
| 08/23/2018 | 08/31/2018 | 1.0 | Just-in-time version | Nyitray |
| 11/21/2019 | 12/08/2019 | 1.2 | Start enrollment of 400 persons in Chicago | Nyitray |
| 01/24/2020 | 02/12/2020 | 3.0 | Enroll Houston version  Houston added to Chicago  Numbers doubled for Houston  Transwomen changed to trans persons  Timeline adjusted | Nyitray |
| 05/15/2020 | 06/09/2020 | 3.5 | Remote consenting instituted due to COVID-19.  Consent ICF changed to reflect changes in the consenting process and subsequent changes to the Pre-Exam and Post-Exam Surveys  Consent goal increased to 900 to accommodate no shows between remote consenting and Visit 1.  Minor wording changes to some survey questions  Co-Investigator Dr. Elizabeth Chiao worksite updated.  Insurance requirement for Houston participants removed. | Nyitray |
| 07/02/2020 | 07/23/2020 | 3.5 | Make ICF and SmartForm consistent | Nyitray |
| 09/06/2020 | 09/13/2020 | 3.6 | Recruit through Crofoot appointment confirmation email/texts | Nyitray |
| 10/28/2020 | 11/27/2020 | 3.8 | Change time between Visit 1 and 2 to six months | Nyitray |
| 12/17/2020 | 02/08/2021 | 3.9 | Update visit 2 procedures, recruitment section to include referral program.  Move participant’s anal self-exam (ASE)/ anal companion exam (ACE) at Visit 2 from in-clinic to at-home due to COVID-19. | Nyitray |
| 08/12/2021 | 02/14/2022 | 4.0 | Add clinic site in Houston  Dr. Chiao transitions to MD Anderson  Thomas Street Health Center added as clinic site in Houston under purview of MD Anderson,  Remove some clinicians’ names and replace with text that does not need to be altered when clinicians change. | Nyitray |
| 03/07/2022 | 05/15/2022 | 4.1 | Send participants notice of ANCHOR Study results  Revise Protocol Change History table to add Date Approved column and modify Revisions column text to better identify amendment  Remove medical records review activity. | Nyitray |
| 05/25/2022 | 05/15/2022 | 4.2 | Increased number to take eligibility survey to 2500. | Nyitray |
| 11/02/2022 | 11/12/2022 | 4.3 | This version accompanies Amendment 26160 which increases the number in the PROSmartForm of those who will take the eligibility survey to 3500 and increases the number to consent to 1050. Number to screen and number to consent do not appear in this protocol; thus no change is made to the protocol. | Nyitray |
| 3/10/2023 | 05/04/2023 | 4.4 | Sending new survey to PAC Palp participants | Nyitray |
| 10/16/2023 | 12/19/2023 | 4.5 | Add MUSC as getting a de-identified dataset | Nyitray |

# LIST OF ABBREVIATIONS

ACE Anal Companion Exam

ASE Anal Self-Exam

CAB Community Advisory Board

CASI Computer-Assisted Self-Interview

CE Cost-effectiveness

DARE Digital Anal Rectal Exam

HRA High-Resolution Anoscopy

HPV Human Papillomavirus

LGBT Lesbian, Gay, Bisexual, and Transgender

MCW Medical College of Wisconsin

MSM Men Who Have Sex with Men

PAC Prevent Anal Cancer

TSM Transgender Persons Who Have Sex with Men

# SITES PARTICIPATING IN THE STUDY

1. Medical College of Wisconsin in Milwaukee will act as the lead site of the study and coordinate the PAC Study clinics for Visit 1 and Visit 2 in Houston.
2. University of Chicago will provide clinical guidance and advice, recruitment, and coordinate and conduct the PAC Study clinics for Visit 1 and Visit 2 in Chicago.
3. Crofoot Clinic will provide clinical guidance and advice, clinic space, education and health care provider exams for PAC Study clinics at Visit 1 and Visit 2 in Houston.
4. University of Texas Health Sciences Center School of Public Health will provide cost-effectiveness analysis, development of a qualitative interview, and recruitment in Houston.

1. The University of Texas MD Anderson Cancer Center will provide clinical guidance and advice and a weekly study clinic at Thomas Street Health Center, conducting the same activities as Crofoot Clinic but under MD Anderson IRB purview.

# PROTOCOL ROSTER

Principal Investigator:

Alan G. Nyitray, Ph.D.
Center for AIDS Intervention Research and Clinical Cancer Center

Medical College of Wisconsin

8701 Watertown Plank Road, Ste. 5400

Milwaukee, WI, 53226

Tel: 414-805-3312
Email: anyitray@mcw.edu

Role: Epidemiologist

Co-Investigators:

Elizabeth Chiao, MD, MPH Medical Oncologist

John Schneider, MD, MPH Infectious Disease Specialist

Aniruddha Hazra, MD Infectious Disease Specialist

Ashish Deshmukh, PhD, MPH Cost-Effectiveness and Decision Scientist

Michael Swartz, PhD Biostatistician

Michael Wilkerson, PhD Behavioral Scientist

Lu-Yu Hwang, MD Infectious Disease Specialist

# STUDY SYNOPSIS

This is a clinical trial of the sensitivity and specificity of anal self-exams and anal companion exams to detect early invasive anal cancer among Chicago and Houston HIV-positive and HIV-negative men who have sex with men (MSM) and transgender persons who have sex with men (TSM).

# MULTI-CENTER STUDY

Medical College of Wisconsin (MCW) is the lead agency in the study. MCW will coordinate activities among study sites and receive all study data. Data will be coded, collected and shared with co-Investigators using REDCap (Vanderbilt University).

1. The University of Chicago (UChicago) staff/faculty will have participant contact during recruitment and when participants come for clinic visits at UChicago.
2. Staff of the Crofoot Clinic in Houston will have participant contact during recruitment and when participants come for clinic visits at Crofoot Clinic.
3. Staff/faculty of The University of Texas Health Science Center at Houston School of Public Health (UTHealth) will have participant contact during recruitment, when participants come for clinic visits at Crofoot Clinic and when analyzing qualitative data.
4. Staff/faculty of MD Anderson will have participant contact during recruitment and when participants come for clinic visits at Crofoot Clinic or Thomas Street Health Center and when faculty view study procedures at the Crofoot Clinic for quality control purposes.
5. MCW staff/faculty will have participant contact during recruitment and when participants come for clinic visits at UChicago, the Crofoot Clinic, and Thomas Street Health Center.

All study data will be collected and sent to MCW which will aggregate and analyze the data to complete Aims 1 and 2. UTHealth will analyze these same data for completion of Aim 3.

## BACKGROUND

Anal cancer is a common cancer among gay, bisexual and other men having sex with men (MSM).^1,2^ Its annual incidence is approximately 50-fold and 5-fold higher among HIV-positive MSM and HIV-negative MSM, respectively, compared to the rest of the general population.^2^ Like cervical cancer, it is primarily caused by human papillomaviruses (HPV).^3^ Unlike cervical cancer, there is no efficacious treatment for anal precancers as there is for cervical precancers.^4^ To date, randomized clinical trials have failed to find an efficacious treatment although other trials continue;^4-7^ thus, professional societies recommend an annual Digital Anal Rectal Exam (DARE) for detection of anal abnormalities, including small tumors, among all MSM.^8-11^ But DARE is underutilized, e.g., most persons, regardless of HIV do not receive an annual DARE^12,13^ and most HIV physicians do not perform the exam.^14^ Therefore, alternative methods for early detection are needed because early detection of small tumors is associated with >85% 5-year survival.^15-17^

To increase detection of anal canal tumors, our NCI-funded phase II pilot study in Houston, Texas determined it was feasible to teach MSM to palpate their own, or their companion’s, anal canal.^18^ Acceptability was 94% among 148 individuals doing the exam on themselves (anal self-exam, ASE) and 92% among 52 couples doing the exam on their companion (anal companion exam, ACE). There was 93% concordance between the clinician’s gold-standard DARE and the participants’ ASE or ACE for any anal canal abnormality.

# STUDY PURPOSE

The long-term goal of the Prevent Anal Cancer (PAC) Palpation Study is to decrease morbidity and mortality from anal cancer by increasing detection of anal canal tumors through self- or partner-palpation of the anal canal.

Having established feasibility among MSM to detect an anal abnormality, next steps are to derive robust estimates of accuracy for both ASE and ACE (e.g., increased sample size in the current study), assess if another clinic can replicate the R21 finding (i.e., UChicago), and assess cost-effectiveness of these exams. In this protocol, “accuracy” is a general term for test operating performance and predictive value. It includes our outcomes of concordance, sensitivity, specificity, positive predictive value, and negative predictive value.^19^

Our central hypothesis is that both ASE and ACE at Visit 1 will have ≥70% sensitivity and ≥90% specificity compared to the clinician DARE gold standard; thus, we believe this study will demonstrate that MSM can recognize when disease is present. We will test the hypothesis with three specific aims:

**AIMS**

Aim 1**: Estimate ASE and ACE sensitivity and specificity.**

Hypothesis: ASE and ACE sensitivity will be ≥ 70% and ≥78%, respectively, at Visit 1; specificity will be ≥ 90% on both exams, and there will be no difference in ASE sensitivity between Houston and Chicago clinics.

Objective: Our objective in this aim is to compare the men’s ASE or ACE result with the clinician gold-standard DARE to determine sensitivity and specificity for each exam at two time points.

Aim 2**: Determine independent factors associated with ASE and ACE concordance.**

Hypothesis: We postulate that practicing the ASE or ACE between visits will be associated with concordance between the ASE/ACE and DARE at Visit 2.

Objective: Using regression analysis, determine factors associated with ASE and ACE concordance.

Aim 3**: Determine the impact of ASE, ACE, and DARE on survival and quality of life, and evaluate the cost-effectiveness of these strategies among HIV-positive and HIV-negative MSM.**

Hypothesis: We hypothesize that initiating training to perform annual ASE or ACE as early as age 30 will lead to cost-effective use of health care resources and will have long-term health benefits relative to no ASE or ACE.

Objective: Complete an economic and survival analysis of ASE, ACE, and DARE using mathematical modeling.

Exploratory aim: Collect qualitative data at Visit 1 from all persons with a false-positive, false-negative, or true-positive result and a 5% random sample of true-negatives to explore participants’ feelings about the ASE/ACE including anxiety among persons with discordant results and any needed support.

# OUTCOMES

Our expected outcomes for aims 1 and 2 are sensitivity, specificity and identification of clinical and behavioral factors associated with concordance. The outcomes of aim 3 will be the computation of incremental cost-effectiveness ratios and net monetary benefits to assess the economic viability of ASE, ACE, and DARE. Data on these outcomes are necessary to provide options for anal cancer screening given underutilization of DARE, no proven precancer treatment efficacy, poor infrastructure for a Pap cytology-based precancer screening protocol, and stigma-related barriers that delay clinic attendance for anogenital examinations. Data from the proposed study could propel the development of a patient-centered intervention for rapid dissemination to a population with extremely high anal cancer incidence and no currently proven screening options.

# SAMPLE SIZE

800 participants completing Visit 1: 400 in Chicago and 400 in Houston

# POPULATION

We will recruit MSM and TSM, aged 25 years and over, who acknowledge sex with men in the last five years or have a minority sexual orientation, have no unresolved health care provider’s diagnosis of anal condyloma, hemorrhoids or anal cancer, speak either English or Spanish, have not had a DARE in the prior 3 months, have no plans to move in the following 6 months, and have access to medical care for any needed referral and treatment that may be discovered during the study.

# DURATION OF PARTICIPANT ACTIVITIES

6 months

| **Study Timeline** | | | | | | | | | | | | | | | | | | | | |
| --- | --- | --- | --- | --- | --- | --- | --- | --- | --- | --- | --- | --- | --- | --- | --- | --- | --- | --- | --- | --- |
|  | 2018 | | 2019 | | | | 2020 | | | | 2021 | | | | 2022 | | | | 2023 | |
| Quarter | q3 | q4 | q1 | q2 | q3 | q4 | q1 | q2 | q3 | q4 | q1 | q2 | q3 | q4 | q1 | q2 | q3 | q4 | q1 | q2 |
| Human subjects approvals |  |  |  |  |  |  |  |  |  |  |  |  |  |  |  |  |  |  |  |  |
| Initiate/meet with CABs |  |  |  |  |  |  |  |  |  |  |  |  |  |  |  |  |  |  |  |  |
| Complete protocol/build CE models |  |  |  |  |  |  |  |  |  |  |  |  |  |  |  |  |  |  |  |  |
| Pilot full protocol & adjust |  |  |  |  |  |  |  |  |  |  |  |  |  |  |  |  |  |  |  |  |
| Train clinicians and observe |  |  |  |  |  |  |  |  |  |  |  |  |  |  |  |  |  |  |  |  |
| Visit 1 clinics |  |  |  |  |  |  |  |  |  |  |  |  |  |  |  |  |  |  |  |  |
| Interim ASE/ACE practice reminders |  |  |  |  |  |  |  |  |  |  |  |  |  |  |  |  |  |  |  |  |
| Visit 2 clinics |  |  |  |  |  |  |  |  |  |  |  |  |  |  |  |  |  |  |  |  |
| Data cleaning & analyses (Aims 1, 2) |  |  |  |  |  |  |  |  |  |  |  |  |  |  |  |  |  |  |  |  |
| CE analyses (Aim 3) |  |  |  |  |  |  |  |  |  |  |  |  |  |  |  |  |  |  |  |  |
| Submit abstracts & manuscripts |  |  |  |  |  |  |  |  |  |  |  |  |  |  |  |  |  |  |  |  |

# PARTICIPANT ENROLLMENT

Participants in each city must meet all stated eligibility criteria.

## Inclusion Criteria

- Be ≥25 years old
- Acknowledge sex with men in the past five years or identify with a minority sexual orientation
- Identify as a male or transgender person who has sex with men
- Spanish or English speakers/readers
- Is HIV-positive or HIV-negative
- Is HPV vaccinated or unvaccinated

## Exclusion Criteria

- Have a primary residence that is not in the Chicago or Houston metro areas
- Have had a DARE in the prior 3 months
- Have unresolved health care provider’s diagnosis of anal condyloma, hemorrhoids or anal cancer
- Have plans to move in the following 6 months
- Is unable to give informed consent

## Recruitment Procedures and Setting

Chicago and Houston staff will use five strategies:

1. Social media advertisements to reach persons not accessing clinics and thus more vulnerable to late presentation of disease (e.g., Growlr, Scruff, Jack’d, and Facebook);
2. Face-to-face encounters in clinics serving HIV-positive and HIV-negative persons;
3. Communication that markets the study within appointment confirmation emails/texts sent by Crofoot Clinic (Houston only)
4. Telephone calls to clinic patients;
5. CAB-associated peer leader recruitment of friends;
6. Pamphlet and flyer distribution at clinic sites and in businesses (e.g., bars), non-profits, and LGBT events.
7. Referral Program

The PAC Referral Program will help direct qualified candidates to the study by incentivizing participants to refer partners, friends and family to be a part of the PAC Study. Specifically:

1. Study staff will provide already-consented participants with information about the referral program:
   1. The referral program is designed to spread the word about PAC Study recruitment to men who have sex with men and transgender persons who have sex with men.
   2. The referral program and the study are entirely voluntary.
   3. If study participants want to help recruit, they can pass along up to five coupons to their partners, friends and family.
2. Each coupon is stamped with a unique number. Staff members will record which coupon numbers were given to which participants.
3. Participants can distribute a digital image or hard copy of the coupons to refer persons to the PAC Study Eligibility Survey in REDCap.
4. The referred persons then engage in study activities as anyone else with the exception that they will provide the coupon code to a staff person when attending a consenting session. Study team members will ask ‘how did you hear about us?’ or ‘who can we thank for referring you to the study?”
5. The participant who referred the person and provided a coupon to person will receive $20 for each coupon redeemed up to a maximum of five coupons or a total of $100. Form of payment, e.g., debit card, gift card or cash app, is up to local site. Sites may decide to pay the referrer after each coupon is received or at regular intervals (e.g., bimonthly).

The study will recruit individuals in each city over 27 months. We will record data on recruitment activities, number of calls and website hits, number who decline, ineligibles, and those not keeping appointments.

Houston. In 2015, 2,084,981 males, aged 25-79 lived in the Houston metropolitan area. If 6.3% of these are MSM, then more than 130,000 MSM live in the metro area. Key recruitment sites are the Crofoot Clinic (i.e., the primary clinic site in Houston), Thomas Street Health Center (TSHC), Legacy Community Health Services (all of which are neighborhood clinics with annual caseloads from 2495 to 5000 MSM), the Montrose Center with multiple projects serving lesbian, gay, bisexual, and transgender people (LGBT), and LGBT businesses and non-profits.

Chicago. In 2015, 3,073,639 males, aged 25-79 lived in the Chicago metropolitan area. If 6.8% of these are MSM, then more than 200,000 MSM live in the metro area. Key recruitment sites are the Chicago Center for HIV Elimination (over 1500 MSM visits in 2014-2015), Connect Health (a sexual health clinic housed in the Infectious Disease department at UChicago, the UChicago HIV prevention and treatment clinic, and two Federally Qualified Health Centers where UChicago providers see patients for HIV treatment and prevention. These sites serve a high proportion of African-American MSM on Chicago’s South Side.

## Community Advisory Board

In both Houston and Chicago, we will rely on a 10-member community advisory board (CAB) in each city for overall guidance. Comprised of both HIV-positive and HIV-negative MSM and TSM and agency representatives who serve them, we will partner with the CAB to ensure study materials are culturally competent for middle-aged, older, and racial and ethnic minority MSM and TSM. After training on study objectives, anal cancer, and the pilot study, the CABs will provide guidance on culturally competent recruitment, educational material design, participant follow-up, and results interpretation. The CAB in Houston will convene for an inaugural dinner during the first 6 months with regular meetings thereafter. The CAB in Chicago already exists and is ongoing.

## Eligibility Procedures

Interested persons will click on a link in recruitment adds or material (or scan a QR code). Persons may also act on recruitment material with telephone calls to study staff. The link will take them to a short description of the study in REDCap and then, if interest remains, a short survey will assess eligibility. Once eligibility is determined, the person will be asked to provide name, email, and phone number in REDCap. Once the person has submitted initial contact information, REDCap will notify study staff in the person’s city that a prospective participant has been deemed eligible and has provided contact information.

**Remote Consenting Appointment**

Staff will go into REDCap to get the contact information and then contact the person to set up a virtual appointment for remote consenting through an electronic meeting like WebEx or a smart phone call (see detailed consenting procedure below). For persons without this technology, face-to-face consenting will be an option.

Once consenting is complete, staff will collect other contact information (e.g., address, alternate email and phone numbers). Then, staff will set an appointment for Visit 1. The person will be provided directions and asked not to have receptive anal sex for 24 hours before the scheduled visit and not to douche or do extra cleaning of the anus on the day of the appointment. Persons will also be asked to trim their fingernails short.

To end the Remote Consenting Appointment, participants will complete an online Pre-Exam Survey in either English or Spanish (all public-facing material will be bilingual in Houston and only in English in Chicago) in REDCap. The Pre-Exam survey will collect the person’s demographics, co-morbid conditions, and EQ-5D quality of life data, in addition to, knowledge, attitudes, beliefs and behaviors regarding anal cancer, self-examinations, sexuality, and COVID-19.

Once the participant clicks “Submit” for the survey, the Remote Consenting Appointment ends.

**Visit 1**

Before the Visit 1 appointment, reminder emails/texts/calls will be made. Eligibility can be done in person at Visit 1 if preferred for persons calling rather than using the online eligibility survey.

## Visit 1 Overview

With identical protocols in Houston and Chicago, persons will receive a short didactic presentation on anal anatomy, practice on pelvic mannequins, and get instructions on doing an ASE or ACE (figure 1). The clinician will then swab the anal canal of each participant, and perform a DARE, without disclosing the results, before leaving participants in private to perform their ASE or ACE. Participants will record their results (as either “normal” or “abnormal”) and then get the clinician’s DARE result along with any needed referrals; thus, the participant result will be judged against the clinician gold standard. Participants will then complete the post-exam computer-assisted self-interview. At the end of Visit 1, persons will be randomized to a condition of encouraged practice of the ASE (or ACE) or a condition of neither encouragement nor discouragement from practice. Persons will have Visit 2 in 6 months when concordance of self- and companion exam with a clinician DARE will again be assessed. Data collected during these visits will be analyzed to complete the aims.

Coordinator provides anal cancer education,

mannequin practice, and ASE/ACE instruction

Clinician performs DARE, without providing results

Individuals perform ASE and partners perform ACE, and record results

Participants complete post-exam CASI,

receive DARE result/safety check from clinician,

and then are randomized

**Figure 1.**

Participants neither encouraged nor discouraged to practice

Participants encouraged to practice

Participants conduct ASE/ACE 24hrs before visit

Clinician provides DARE and DARE result; participants complete post-exam CASI

**At Visit 2**

**The Day Before Visit 2**

## Visit 1 Detail

After assessment for COVID-19 symptoms/signs, participant weight, height, and waist circumference will be measured followed by rooming the participant with 1-3 other persons in the training room. For this group, the study coordinator will provide anal anatomy/disease education and ASE/ACE instruction. The study coordinator will use pelvic mannequin 1 which has a healthy anal canal and mannequin 2 with a 0.6 cm x 1.0 cm tumor on the anal canal wall (Kyoto Kagaku Co., Kyoto, Japan). The study coordinator will demonstrate on the mannequins how to palpate for an anal canal tumor and ask each participant to practice on the mannequins and then identify the mannequin with the tumor. The study coordinator will then review the ASE/ACE pictorial instructions with participants. Key elements of ASE and ACE education are first viewing the perianus region (with a mirror or phone selfie), finding a comfortable position, and making sure the full 360º of the anal canal is palpated.

In brief, the study coordinator will describe a 5-step ASE/ACE: 1) view the perianus, 2) after gloving and adding lube, touch the anal opening and feel for an abnormality, 3) put a finger in to the first knuckle, try to feel 360° around for an abnormality, and then push in to the second knuckle and feel again, 4) pull finger out and then re-insert to perform the procedure again, and 5) (for individuals only) switch hands (or fingers) and repeat to feel 360° of the canal. The full 360° palpation will be emphasized when practicing on the mannequin by showing the participant that the full circumference is hard to palpate with only one finger. Participants will likely need to switch hands although it may be possible to use the index finger first and then opposable thumb on the same hand to palpate the full anal canal. The manner used by the participant is less important than full palpation of the anal canal. The study coordinator will emphasize that it is not important to distinguish between diseases: any abnormality should be noted. This will end the training session.

After each participant is put in a private exam room, a highly-experienced clinician will collect anal canal exfoliated cells from the participant using a swab. The clinician will follow the Darragh swabbing protocol (e.g., twirling the swab and slowly removing the swab while counting slowly to ten, and applying pressure to the anal canal walls).^20^ The clinician will insert the swab into a prelabeled vial with 2 mL of STM, write the date on the vial, and then place the vial in a cooler. Onsite processing of the specimen will occur at the end of each clinic which consists of logging the specimen in REDCap, vortexing for 45 seconds and then aliquoting the liquid into 3 cryovials that are then stored in a -80C freezer. On a monthly basis, the study coordinator will overnight specimens in styrofoam containers to the Medical College of Wisconsin Tissue Bank laboratory where each specimen will be recorded in a logbook with participant ID# and date. These specimens will be archived and made available for testing of candidate molecular biomarkers. These results may provide further information on the role of ASE, ACE, and DARE in detecting anal abnormalities. The PI will observe the swabbing protocol of the first 5 participants in each clinical site and re-observe on a quarterly basis.

After collection of the anal canal exfoliated cells, the clinician will then perform a DARE without disclosing results to the participant; however, the clinician will describe to the participant the process of the DARE as the DARE is performed (e.g., “I am first looking at the area around your anus to see if there is anything that doesn’t look normal.” And “I am now feeling all the way around your anal canal to make sure I don’t miss any part of it.”). The clinician will be trained to not provide inadvertent cues on DARE results. After completing the DARE, the clinician will then leave the exam room and record the DARE results for both perianus and anal canal according to published guidance.^21^ If an abnormality was observed, the clinician will note its location, size in diameter, appearance and color (if perianal) and other notable characteristics. The duration of the anal canal swabbing and DARE will be noted to inform cost-effectiveness analysis.

The first 40 persons in each city will receive a second DARE done by another experienced clinician immediately after the first DARE done by the primary clinician. The clinicians will be kept separate for the two DAREs and not disclose their findings to each other. The second DARE is done to assess the reliability and repeatability of the primary clinician’s DARE.

Once the clinician leaves the exam room, participants will remain and conduct the ASE/ACE. Participants will be trained to stop the procedure if pain is felt. The participant will record on a form the ASE/ACE result: either normal or abnormal. The participant will inform staff that the ASE/ACE is complete. If the participant detects an abnormality, staff will ask if the abnormality was at the perianus and/or anal canal, what it felt like, (i.e., hard or soft) and its size (using varying sizes of circles on paper). Persons will then complete a Post-Exam CASI in the exam room (one member of a couple will do the CASI in another room to give each participant privacy). The couples’ protocol mirrors the individuals’ protocol except that each partner will ask the other for consent before performing the ACE. Prostate and distal rectal palpation will neither be taught nor expected.

The Visit 1 post-exam CASI requires ~20 minutes to complete and asks about ASE/ACE acceptability and self-efficacy, in addition to intentions-to-seek subsequent care, body positioning for the exam, history of anal self-exams, alcohol use, recent sexual behavior (type and frequency), and anogenital hygiene. Questions to support cost effectiveness analysis are included.^22^

While the participant completes the post-exam CASI, the study coordinator will use REDCap to randomize the participant (Arm 1 – practice or Arm 2 – control); if the person is a true negative (the coordinator will possess both participant and clinician results at this point) the study coordinator will check REDCap to determine if this true negative will receive a qualitative interview.

After the post-exam CASI, participants will remain in the exam room (couples will be reunited) and receive DARE results. If the screening result is a true negative (either for an individual, or, for couples, both partners), the study coordinator will provide the results, e.g., “You reported that your anus felt and looked normal and that’s what the clinician said too. So now you know what your anus is like when it’s completely normal. If something changes, you’ll be better able to notice there’s a difference.” If the result is a true positive, false positive or false negative, then the clinician will provide the result.

If there is a finding of an abnormality on DARE, then standard clinical practices will be followed. If needed, clinicians will treat limited external disease, triage other disease, and ask the participant to call if there are any concerns in the following week. If the result is a false positive or false negative, the clinician will request to immediately perform a second DARE for adjudication of the discordant result. All participants with referrals due to abnormal DAREs will be followed to assess care-seeking behavior and outcome.

An exploratory aim of this study is to collect qualitative data at Visit 1 from all persons with a false-positive, false-negative, or true-positive result and a 5% random sample of true-negatives (~65 persons in each city based on pilot data, or ~1 per clinic week over 2 years). Each will be invited to participate in an individual interview of up to 15-minutes conducted by a member of the project team at each clinical site. Interviews will explore anxiety among persons with discordant results and any needed support. Faculty will train staff to use a semi-structured guide for interviews.

## Randomization

At the end of Visit 1, all participants will be randomized to one of two conditions: Arm 1 (practice) or Arm 2 (no practice). One-half of participants in each city will be randomly selected and encouraged to practice the ASE/ACE 3 months before Visit 2. Persons randomized to the control arm will neither be encouraged nor discouraged from practicing the ASE/ACE.

## Arm 1 - Practice Arm

Persons in the practice arm will pick a date three months after Visit 1. The study coordinator will record the date and provide participants written ASE/ACE instructions. Participants who report more than minimal pain associated with ASE/ACE at Visit 1, or otherwise have contraindications based on DARE results, will be asked to not to practice if randomized to the practice Arm. However, all persons will be asked to attend Visit 2 to engage in study activities that are not contraindicated at the time of Visit 2. Participants will be reminded that we will schedule a Visit 2 appointment and that we will also contact them in 3 months and periodically to keep in touch to support study retention.

## Arm 2 – Non-practice Arm

Persons in the non-practice arm will be reminded that we will schedule a Visit 2 appointment and that we will also contact them periodically to keep in touch to support study retention. Persons in the non-practice arm will be neither encouraged to practice nor discouraged from practice. If asked about practice, study staff will reply that the participant is not expected to practice since the ASE/ACE is not known at this time to be beneficial. The PAC Palpation Study is trying to determine if it’s beneficial.

After end-of-Visit 1 study staff will provide a $50 incentive and also tell the participants about the Referral Program for recruitment. Anticipated visit duration is about 75 and 90 minutes for individuals and couples, respectively.

## Between Visit 1 and Visit 2 study procedures

Arm 1 – Practice.

*If participant had a normal DARE at Visit 1*, OR *if participant had an abnormal DARE at Visit 1 that required no referral (e.g., a scar or non-prolapsed hemorrhoids)*, study staff will make reminder calls (or emails/texts) and follow-up calls for the scheduled practice session. At the follow-up calls, study staff will record the participant’s result for the self/companion exam and the level of anxiety and pain, if any, associated with the exam. If the participant reports more than minimal pain and anxiety, they will be asked to return to the clinic for an HCP exam.

If a new abnormality is detected by the participant, i.e., an abnormality not recorded at Visit 1, the study coordinator will request they return to the study clinic site for a clinical examination. While requesting they return to the clinic, staff will record the participant description of the abnormality, and their level of anxiety and pain associated with the ASE/ACE practice. When the participant returns to the clinic, staff will record medical outcomes for each person.

Participants not wanting to return to the clinic will be provided referrals by the health care provider at the clinic. Efforts will be made to contact persons once each week for up to four weeks to encourage a return to the study clinic or completing of the referral elsewhere.

*If participant had an abnormal DARE at Visit 1 that required referral (e.g., condyloma or suspicious lesion)*, the person can still participate in practice unless the clinician advises that the ASE/ACE is contraindicated due to the Visit 1 abnormality, or the person reported more than minimal anxiety or pain due to the ASE/ACE at Visit 1. During follow up, staff will ask about referral completion, resolution of the abnormality, and practice ASE/ACE results. If referral has still not been completed, staff will again encourage completion of referral or coming back to an upcoming study clinic for another DARE.

Participant activity and results between Visit 1 and Visit 2 will be documented in REDCap.

## Preparation for Visit 2

Two weeks before the six-month anniversary of Visit 1, staff will contact participant to confirm the Visit 2 appointment time, give instructions for the pre-visit ASE/ACE, and how to report the result. Regardless of prior results, all persons will be asked to return for Visit 2. Persons with a prior contraindication for ASE/ACE will not be asked to complete the Visit 2 ASE/ACE until cleared by a clinician. To reduce loss to follow up, staff will contact participants two days before Visit 2 to encourage attendance.

Participants will complete the Visit 2 pre-exam computer-assisted self-interview before coming to the clinic for Visit 2. The Visit 2 pre-exam CASI will add questions to the Visit 1 pre-exam CASI to assess 1) knowledge retention about how to do an ASE/ACE, 2) use of ASE/ACE between visits, 3) health-seeking behavior if a participant detected an abnormality between visits,4) social desirability bias^23^ (to assess response bias related to being compliant about practicing), and social support questions.

After the pre-exam CASI, participants will be asked to do the ASE/ACE at home within 24 hours before the Visit 2 in-clinic visit. ASE/ACE instructions will be provided upon request. The person will then complete an ASE/ACE, record results as either normal or abnormal, and text the result to the study staff as soon as they’re done with the anal exam. Persons in the control arm will be asked about performing the ASE/ACE between visits, technique, and frequency to control for potential confounding. Moving the ASE/ACE to a home-based exam will shorten the participant’s time in the clinic which, regarding nosocomial infections like coronavirus, supports a safer clinic visit for participants and may encourage more participants to attend the visit.

Participants will complete an online COVID-19 symptoms survey within 24 hours of Visit 2.

## Visit 2 Study Procedures

During Visit 2, study staff will confirm the participant’s ID, measure their weight and waist circumference, and then room the participant in a private room.

The clinician will collect an anal canal swab using a procedure identical to Visit 1 and provide a DARE. The clinician will immediately give DARE results (with results recorded using the same form from Visit 1) before participants take the post-exam CASI which will explore participant responses to knowledge of false-positive, false-negative, true-positive and true-negative results, e.g., how does a false-positive result impact intention to perform the procedure in the future?

After completion of the post-exam CASI, persons will be paid $80. They then can leave.

If the health care provider found an abnormality at Visit 2 during DARE that required referral, we will maintain contact with the person to record health seeking behavior, treatment received, if any, and the outcome.

Persons who initially entered the study with a partner, but are no longer partnered at Visit 2, can switch from an ACE to an ASE. These changes will be noted and accounted for in analyses. Persons who conducted an ASE at Visit 1 will be asked to conduct an ASE at Visit 2.

To determine the proportion of consented persons who would be interested in a follow-up anal cancer study, all persons who consented to the parent study using a UChicago ICF or an MCW ICF will be contacted near study end. Persons will be contacted by the email or phone number provided by them in the parent study. If necessary, non-respondents will be contacted up to 3 times starting with their preferred method of contact for the first two contacts and switching to another method for the third contact. After asking the one question in this effort (see Renewal Survey file), we will record a Yes or No in the appropriate REDCap field to record their interest in a future study.

## Data Collected from Study Participants

For all data collected in the study, personal health information in items 1-6 below will be kept at the Medical College of Wisconsin (MCW) on a secure internet-based server that meets Health Insurance Portability and Accountability Act privacy standards for health records.

1. For each participant, the study coordinator will document completion of study forms and activities (e.g., consents and anal swabbing) and record height, weight, and waist circumference.
2. The health care provider will record on hard copy the results of DARE on participants. These results may be retained in the clinic and then extracted from the participant record by study staff.
3. Participants will use a one-page form in the clinic to record their detection or non-detection of perianal and anal canal abnormalities at Visit 1. Participant will text their result to a staff member after completing the in-home ASE/ACE.
4. The study coordinator will record the participant’s understanding about the location and size of the abnormality.
5. In addition to the eligibility CASI before consenting, participants will complete 4 CASIs during the study, both pre-ASE/ACE/DARE CASIs and post-ASE/ACE/DARE CASIs at each visit.
6. Qualitative interviews will collect some participants’ reactions to the ASE/ACE results at Visit 1, participants’ suggestions on needed resources after doing the ASE/ACE, and suggestions for modification of training.
7. Staff will collect data between Visit 1 and Visit 2, e.g., practice documentation, ASE/ACE results, and referral completion.
8. Participants consenting with a UChicago or MCW ICF will be contacted to determine their interest in a follow-up anal cancer study.

# BURDEN OF TIME ON PARTICIPANTS DOING THE ASE

| **Activity** | **Duration** |
| --- | --- |
| Pre-Visit 1 - Consenting. | 12 min |
| Pre-Exam Online Survey | 8 min |
| **Visit 1** |  |
| 1. Appointment greeting and photo ID check | 1 min |
| 1. Body measurements and COVID-9 symptom check | 3 min |
| 1. Anal anatomy and pathology and self-/companion exam education (Visit 1 only) | 10 min |
| 1. Anal swab | 2 min |
| 1. DARE | 2 min |
| 1. Anal-self exam or Anal companion exam | 10 min |
| 1. Post-Exam CASI | 20 min |
| 1. Results including referral or schedule for treatment if needed | 7 min |
| Total time | 75 min |
| Other: for first ~40 persons in each city, second DARE (QA and reliability study) | 2 min |
| Other: estimated 28 persons in each city will have discordant result and clinician will offer to give participant another DARE to adjudicate | 2 min |
| Other: for an estimated 65 persons in each city, a qualitative interview will be done | 15 min |

Note. Persons in couples doing the ACE at Visit 1 will require about 90 minutes for the visit. For Visit 2, persons will not receive the anal anatomy/pathology and ASE/ACE face-to-face education. Rather, they will be given instructions and asked to do the exam without benefit of a training session with study staff; then the clinician will do a DARE and provide results and any needed referrals during the same face-to face.

|  | |
| --- | --- |
| Activity | Duration |
| Immediately before **Visit 2** |  |
| 1. Pre-Visit 2 Pre-Exam Online Survey | 10 min |
| 1. Anal-self exam or Anal companion exam | 8 min |
| 1. COVID-19 Symptom Online Survey | 3 mins |
|  |  |
| During **Visit 2** |  |
| 1. Appointment greeting, body measurement [weight and waist circumference] ID confirmation | 3 min |
| 1. Anal swab, DARE and results with clinician including referral or schedule for treatment if needed | 5 min |
| 1. Post-Exam CASI | 20 min |
| Total clinic time | 28 min |
| Other: estimated 25 persons will have discordant result and clinician will offer to give participant another DARE to adjudicate |  |

# STATISTICAL ANALYSIS

## Aim 1

The central hypothesis is that both ASE and ACE at Visit 1 will have ≥70% sensitivity and ≥90% specificity using the clinician DARE as the gold standard. We will test our central hypotheses at Visit 1 by comparing ASE and ACE sensitivity and specificity to random guessing (i.e., sensitivity and specificity=50%) using a one-sample test for proportion. We will then conduct a test of equivalence using two, one-sided likelihood score tests to compare ASE sensitivity in Houston vs Chicago clinics to determine if the ASE performance is equivalent in each city; thus, we will assess the homogeneity of Houston and Chicago sensitivity before combining results. We focus on sensitivity since this factor performed worse than specificity in the pilot study and had wide confidence limits. Sensitivity will also be stratified by other factors, for example location (anal canal vs perianal) and co-morbidities, to determine the effect on sensitivity. Tests will be two-sided with a 0.05 alpha. We will estimate percent agreement, positive predictive value, negative predictive value and perform receiver operating characteristic (ROC) curve analysis for a binary outcome.

To test our central hypothesis, our pilot data indicate 1) 11% of persons will have condyloma, hemorrhoids, skin tags, scars, or malignant tumors at Visit 1 at the anal canal or perianal region, and 2) a sensitivity of ≥70% on ASE and 78% on ACE and specificity ≥90% for both at Visit 1. Given a null value for sensitivity and specificity of 50% (since we seek to rule out accuracy due only to chance), our power to detect sensitivity ≥70% among 600 individuals is 0.90 and 0.81 among 200 couples. Power to detect specificity ≥90% among both individuals and couples is >0.90. For multiple comparisons we will use the False Discovery Rate. Using a test of equivalence, our hypothesis of no difference in ASE sensitivity between Houston and Chicago assumes that a difference of less than 0.15 constitutes equivalent sensitivity given that a smaller difference may have little practical relevance for adjusting the protocol in either city. Under this definition of equivalence, we have at least 0.87 power to detect a lack of equivalence in sensitivity between the cities if the actual observed difference ranges from 0 to 0.05. Power was assessed with PASS13.

Qualitative data at Visit 1 will be analyzed with concurrent mixed-methods design, i.e., a participant’s study ID number will link qualitative to quantitative data. Demographic characteristics and summary statistics from the CASIs involving quantitative cognitive and affective responses to the ASE/ACE of the ~125 persons getting a qualitative interview will be imported into Atlas.ti (Scientific Software Development GmbH, Berlin, Germany). During qualitative analysis, the imported quantitative data will be used to stratify the 125 persons by demography or other categorical data. Qualitative data will be analyzed as it is collected, per the constant-comparative method.^24^ Specifically, as interviews are completed, transcripts will be entered into Atlas.ti for analysis within ~72 hours. Faculty and a graduate research assistant team will independently code the data, meet to compare codes, and resolve discrepancies by collapsing and reorganizing codes to arrive at a common codebook. The researchers will then use axial coding to identify relationships between codes. To arrive at an explanatory model, the team will compare findings and recommendations to the existing literature,^18,25^ and consult CAB members for interpretation. The final explanatory model will aid in understanding how anal cancer knowledge and perceived risk for anal cancer affects ASE/ACE performance. In particular, we will explore anxiety among persons with false-positive results because the false-positivity rate was 14% among persons in the pilot study who said anal cancer concerned them “a lot,” while among persons who had no, little, or some concern, the false positivity rate was 5% (*p*=0.58) suggesting that anxiety may play a role in false-positive results.

## Aim 2

There is one primary outcome for Aim 2: the association between practice and concordance at Visit 2. Concordance is binary and equals 1 when both participant and clinician identify an abnormality or agree on its absence; thus, the statistical analysis is premised on detecting an anal abnormality, not a cancer. We postulate that practicing the ASE or ACE between visits will be associated with ASE/ACE and DARE concordance at Visit 2. To derive unbiased estimates in multivariable modeling for the association between practice and concordance, we will use directed acyclic graphs to identify potential confounders to include in models (e.g., age, ethnicity, race, city, anxiety, chronic disease, and, for ACE, co-teaching and reported familiarity with a companion’s body) and intermediate variables to exclude from multivariate modeling; however, given successful randomization, bias, including confounding, should be minimized. Both intent-to-treat and per-protocol analyses will be conducted (we expect a similar proportion of persons in the practice and non-practice arms to switch arms of their own accord, i.e., crossover, between visits). The association will be assessed with logistic regression. After univariate analyses, variables where p≤0.25, and which are not intermediate variables, will be added to a multivariable model using backward selection with binary practice (yes/no) retained in the model. After adjustment for potential confounders, factors where p<0.05 (two-sided) will be considered statistically significant. Adjusted and un-adjusted odds ratios will be reported with 95% confidence intervals. We will use a similar approach to derive unbiased estimates in multivariable modeling for the association between continuous waist size and concordance; however, we will also use ROC curves to ascertain the potential for a threshold effect of waist circumference size on concordance. We will also assess the full set of other study data and their potential associations with concordance. For multiple comparisons we will use the False Discovery Rate. Finally, we will investigate effect modification by age and individual/couple status.

For the Aim 2 hypothesis, that practice will be associated with concordance in all persons at Visit 2, sample size will be adjusted downward by dropouts and non-resolving abnormalities from Visit 1. We expect 11% of persons (n=88) to have an abnormality at Visit 1 (based on pilot data) with most of these being hemorrhoids and condyloma. We conservatively expect 50% of hemorrhoids and condyloma to resolve in 9 months; thus, there will be a minimum of 44 non-resolving events from Visit 1 and another 10% of persons will dropout (800-44=756; 756-76 = 680). We have 80% power to detect an odds ratio of at least 1.55 or larger if concordance is 85% and n=680.

## Aim 3

All data used in Aim 3 come from literature, our previously published studies, and data collected in Aim 2 and Aim 3. For example, CASI and DARE results will provide data on screening test characteristics, screening test costs, and quality of life scores. The parameters required to structure the decision-analytic model will be derived from these sources.

For comparative cost-effectiveness analysis, we will consider these strategies: no screening, ASE, ACE, and clinician DARE. In addition, we will evaluate the optimal interval for performing these tests (annual, biennial, or triennial) and optimal age to initiate training MSM to perform the test.

For the analysis, we will develop an individual-level state-transition (i.e., micro-simulation) model (STM). They also enable screening interval evaluation, and appropriate screening ages. The model will be programmed in TreeAge Pro 2016 software (TreeAge Software, Inc., Williamstown, MA).

The starting (hypothetical) cohort will be comprised of MSM in the US distributed in health states based on the baseline prevalence of anal HPV infection and anal dysplasia among HIV-positive persons. Patient profiles will be defined based on history/diagnosis of anal abnormalities, HIV disease status (i.e., current and nadir CD4 count of CD4>500, CD4 200-500, or CD4<200), HPV vaccination status, and receipt of antiretroviral therapy (yes or no). We identified these characteristics as they might impact long-term outcomes including survival, quality-adjusted life years (QALYs), and anal cancer mortality risk. Baseline age will be assigned according to the age distribution of MSM in the US. The cycle length (i.e., the amount of time represented by each model simulation step) will be 12 months which seems appropriate (1) since persons in our pilot study were willing to screen at least every few months, (2) given recommendations of an annual DARE, and (3) given a higher likelihood that more frequent exams will detect anal tumors at an earlier stage.

Health quality of life scores will be assigned for each health state using utility scores (i.e., quality of life weights) ranging from 0.0 to 1.0. This assignment will be computed as the mean EQ-5D score associated with each participant who is classified in each health state.

Direct costs of performing ASE, ACE, and DARE (e.g., cost of gloves and lubricant) will be determined using a “micro-costing” approach with data on resources used coming from Aim 1. Clinician time cost for performing DARE will be obtained from the Medicare fee schedule (using HCPC S code G0102). We will also include direct non-health-related costs, for example, individual and/or couple time cost for performing ASE/ACE (collected in the R21 pilot study). Previously, our group estimated the cost of anal cancer care in the US and clinical and cost-effectiveness of treating early stage anal cancer. We have also identified the costs of care for HIV, condyloma, and hemorrhoids.

The accuracy of our decision-analytic model will be evaluated by performing extensive model validation that will compare model-predictions to the outcomes reported in the literature. For instance, we will compare age-specific cumulative risk of anal cancer among HIV-positive MSM to the cumulative risk reported in a recent analysis of the HIV/AIDS Cancer Match Study. Similarly, we will compare model-predicted mortality among HIV-positive persons to the mortality risk estimated independently using databases from SEER-Medicare (>3,500 HIV-positive men with 33 anal cancer cases) and NA-ACCORD (>100,000 HIV-positive persons (>50% MSM) with 245 squamous cell anal carcinoma cases). We will assume an average DARE utilization rate of 23% in the US. If the predicted model outcomes fall outside the 95% confidence intervals of the reported data, we will calibrate the parameters such that the model outcomes are within the limits.

The microsimulation allows us to perform calculations of the expected economic costs and clinical outcomes of life expectancy and long-term quality of life and estimate stochastic uncertainty with confidence intervals for each strategy. When executed, an individual transitions from one state to another according to the transition probabilities, participant physiologic characteristics, and prior health history. As an individual passes through each health state, the utility value is accumulated for a period of time equal to the cycle length of the microsimulation model (i.e., 12 months). By accumulating utilities over the lifetime of the model, we will obtain the quality-adjusted life expectancy for each strategy. The same methodology applies to determining the expected costs for the strategies. We will discount costs and QALYs by 3%.

Cost-effectiveness using societal- and health care-sector perspectives will be evaluated in terms of incremental cost-effectiveness ratios (i.e., the ratio of difference in costs between 2 alternatives to the difference in effectiveness between the same 2 alternatives), comparing each strategy in sequence from lowest expected cost to most expensive. Issues of strong and weak dominance of clinical strategies will be considered. We will use the commonly accepted willingness-to-pay (WTP) threshold of $100,000/QALY to determine the most-cost-effective strategy. As per the guidelines from the second panel on cost-effectiveness analysis, we will also estimate net monetary benefits (i.e., the linear combination of costs and effects expressed in US dollars). Other long-term health outcomes will be estimated in terms of lifetime risk of developing anal cancer among individuals who performed ASE or ACE relative to no screening, lifetime anal cancer mortality risk among those individuals, and life expectancy gained attributed to screening using anal examinations.

To evaluate robustness of the outcomes, we will perform comprehensive sensitivity analysis on all model parameters. We will perform one-way sensitivity analyses, varying model parameters (e.g., screening accuracy, treatment efficacy, costs, etc.) to look for consistent patterns in the results. Decision uncertainty will be evaluated using probabilistic sensitivity analysis (PSA). For PSA, we will perform 10,000 runs of Monte Carlo simulation using two nested simulation loops where the inner loop will evaluate the outcomes across the simulated population and the outer loop will sample those parameters to reflect the uncertainty in the model parameters. The outcomes of PSA will be presented in the form of a cost-effectiveness acceptability curve, which plots the probability that an intervention is cost-effective against various willingness-to-pay thresholds.

Exploratory analysis of de-identified data may also be done by collaborators including the Medical University of South Carolina. The Safe Harbor method will be used to de-identify the data https://www.hhs.gov/hipaa/for-professionals/privacy/special-topics/de-identification/index.html#standard.

###

### **PROTOCOL FIDELITY AND QUALITY CONTROL**

Protocol activities will be documented with a checklist. To ensure identical protocols in each location, qualified clinicians will conduct clinical exams of participants after being trained in the study protocol by Dr. Nyitray and after being trained in the study's clinical procedures by either Dr. Hazra, Dr. Schneider, Dr. Chiao, or Nurse Practitioner Maggie White.

At study start and semi-annually, the PI will observe all procedures for two individuals in both Houston and Chicago sites and provide critique to prevent drift in study protocol. Houston co-I Dr. Chiao and Chicago co-I Dr. Schneider will attend five initial Visit 1 appointments in Houston and Chicago, respectively, to observe the clinician’s DARE technique and protocol.

To document the repeatability of the NP DARE results (which was 95% in the pilot), a second clinician at each clinic will perform a second DARE on ~40% of persons in each study site in the first six months of enrollment (i.e., 40 in each city). The secondary NPs will also be trained in study protocol and DARE.

## HUMAN PROTECTIONS

## Overview

The principles of Institutional Review Board (IRB) approval and informed consent described in the Food and Drug Administration (FDA) regulations (21 CFR Part 50 and 56) and Department of Health and Human Services (DHHS) regulations for the Protection of Human Subjects regulations (45 CFR Part 46)] must be followed. IRB approval of the protocol and the informed consent form must be given in writing.

The Investigator(s) must receive a copy of the letter of approval from the IRB, which specifically approves the protocol and informed consent, before participant enrollment. The IRB must also approve any significant changes to the protocol and documentation sent to the Investigator(s). The IRB must review the research project at least once every 365 days during the project. Continuing approval of the project must be given in writing and provided to the Investigator(s).

Records of all study review and approval documents must be kept on file by the Investigator and are subject to inspection during or after completion of the study. Adverse events must be reported to the IRB according to local procedures. The IRB should receive notification of completion of the study and final report within 3 months of study completion and termination. The Investigator will maintain an accurate and complete record of all submissions made to the IRB, including a list of all reports and documents submitted. The Investigator will grant monitor(s) and auditor(s) from the NCI and regulatory authorities access to the participants’ data. Participants confidentiality will be maintained and will not be made publicly available to the extent permissible by the applicable laws.

As detailed below

- Persons must have provided informed consent to participate in the study;
- All participant data must be handled and stored securely;
- Study risks will be reduced with adequate protections from risks;
- Participants will be monitored for adverse events;
- A Data and Safety Monitoring Board will monitor participant welfare.

Institutional Review Boards (IRB) at UChicago, UTHealth, and MD Anderson will receive approval for their protocols and consents and then submit those approved documents to the Principal Investigator who will include these documents in an IRB application at MCW. Once MCW has approved the full study protocol, recruitment can begin. All persons working with participants or participant data will have relevant human protections training.

## Informed Consent Process

The process for obtaining informed consent will use a video conference or a phone call to a person with a smart phone. Persons who do not have access to this technology will be consented in person.

At the beginning of the electronic consent meeting (pre-arranged by appointment), staff will ask participant to confirm email address and name and then use REDCap to send the person a link by email or text (depending upon technology used by the person). Person will click on the received link to open the ICF in REDCap. Receipt of the unsigned ICF on the computer/tablet or smart phone, will be confirmed by the participant before beginning the consent session. With delivery of REDCap electronic versions of the ICF, both study staff and participant will now be viewing the study ICF. Study staff will explain study procedures, risks, benefits, confidentiality. Prior to signature, the staff member will ask the participant if they have any questions and, if not, to briefly review their understanding of their participation to be sure the information was clearly understood. The person obtaining consent will emphasize that participation is voluntary and that participants can choose to join or, if they do join, leave the study at any point without any repercussions. Study staff will frequently refer to pages, headings or some other marker in the ICF to make sure the participant is following along.

Persons agreeing to provide consent will sign the ICF as follows: a. Touch screen - persons will use finger or mouse to sign; b. No touch screen - persons will use a mouse to sign. After signing, person will submit by clicking on/touching the “Submit” button on the REDCap screen displaying the ICF. REDCap has two settings to send signed ICFs to participants and both will be employed to allow participants to have their choice of either downloading a pdf of the signed ICF or receiving an email with signed ICF attached.

For consents in person, the participant's identification is checked when coming to the consent face-to-face meeting. When a participant appears for an appointment, they will be thoroughly informed of all study procedures, potential risks, benefits, and that one can discontinue participation in the study at any time without risk to subsequent care at the place(s) where health care is received, etc.

Participants in couples must both consent to the study to be enrolled as partners. They can be consented together.

## Use and Access of Protected Health Information (PHI) and Retention of Records

The primary platform for collection of all study data is REDCap (REDCap, Vanderbilt University) which will store data on a secure internet-based server. REDCap meets Health Insurance Portability and Accountability Act privacy standards for health records. Study PCs will have passwords and strong data encryption installed as per requirements at MCW.

Staff will collect participant contact information such as email address, telephone number, and mailing address to send study reminders and to share study-related updates. Staff will also collect information about secondary contacts to support study retention if initial contact information is no longer valid. Staff will not collect social security numbers.

All other study data will be coded only with a Study ID. Personal identifiers such as name, address and phone number will be removed from clinical results. The computer-assisted self-interviews will be identified only by ID number and do not contain personal identification information. Information linking the participants’ identity to the study code will be kept in a Linking Log on a secure server. Separate cabinets will contain informed consents.

CASI data will be delivered directly from tablets and PCs in Chicago and Houston to MCW servers using REDCap. Clinician and participant data from clinic visits will be collected in hard copy and then entered into REDCap. Qualitative interview data will be collected in hard copy and by audio recordings on study computers. Audio recordings will be transcribed with both recordings and transcriptions stored on MCW servers. Original hard copies of all data will be stored in locked file cabinets separate from linking logs and consents.

After all study queries and analyses are completed, the data/PHI will not be destroyed but will be archived in a secure long-term storage site to keep an accurate record of screened and enrolled subjects for the sponsor and potential audit purposes only specific for this study. No information that could lead to personal identification of participants will be included in any of the reports or given to any non-authorized person. Staff will store the identified information for 10 years after study completion, as required by the NIH. Staff will destroy all personal identifiers after analyses are completed.

## Risks to the Subjects

The risks to participants can be divided into four groups: a) risks during the health care provider DAREs, b) risks during collection of anal canal exfoliated cells, c) risks during the ASE/ACE, and d) concerns about disclosure of confidential, sensitive information.

a) Each health care provider will perform a DARE on the participant which will entail inserting a gloved finger into the anal canal. While this is a common and safe procedure, it may be mildly uncomfortable or embarrassing for some participants.

b) Collection of anal canal cells: Some persons may feel embarrassed about a health care provider inserting a swab into the anal canal. The swab can be uncomfortable, and occasionally there can be some bleeding. Justification: Currently there is no acceptable anal cancer biomarker. These specimens will be archived and then available for testing of candidate biomarkers.

c) The participant will perform an ASE/ACE which will entail the participant looking at the perianal region (individuals will use a mirror or selfie) and then inserting a gloved finger into their own anal canal or that of their partner. While it is common for a person to insert a finger into the anus or the anus of another while having sex or engaging in exploration or self-hygiene, it may be uncomfortable or embarrassing for some participants. Partners will ask each other for consent before performing the procedure. If an anal fissure is present, finger insertion could be more painful. It is also possible that a participant will palpate a mass which may induce anxiety, e.g. if the participant imagines that the mass could be a malignant tumor. This risk is justifiable because the participant will, at the same visit, meet with a health care provider who completes a DARE on the participant and who can explain the results and support onsite treatment or referral for treatment if needed.

d) This study will ask sensitive questions about sexual behavior and disease status. Some people may fear that unauthorized persons, who may discriminate, might obtain this information or otherwise cause a change in the participant’s social status or access to benefits to which participants would otherwise be entitled. In addition, some persons who do a qualitative interview may feel uncomfortable talking about their ASE/ACE experience. This information is needed to better understand how to modify instruction in the procedures to reduce false-negatives and false-positives, and how to support health care engagement for true-positives. The study team will do everything possible to protect subjects’ confidentiality.

## Alternatives to study procedures

The only alternative screening for anal cancer is an anal pap test and high-resolution anoscopy; however, this screening modality is not a standard practice at the current time, nor one widely recommended or available, especially for HIV-negative men who have sex with men who are nevertheless at increased risk for anal disease. The infrastructure for high-resolution anoscopy is limited so that even persons with HIV who have abnormal anal Pap tests may wait months before getting an appointment. This is one reason that expert opinion recommends DARE for HIV-negative and HIV-positive MSM.

## Protections against risk

When the nurse practitioner performs a DARE on the participant, standard medical practice for a DARE will be used including gloving, use of lubricant, and a few seconds of hesitation while the finger is on the anal os to allow sphincter muscles time to relax. The nurse practitioner will visually inspect the perianal region before performing the DARE. If the nurse practitioner sees an anal fissure, anal fistula, or other condition that is a contraindication for a DARE, the nurse practitioner will discontinue the clinical visit and provide treatment and/or referral to the participant. The participant will be allowed to rejoin the study after the condition heals. Confidential, sound-proofed exam rooms are provided for all exams.

When giving DARE results at the end of the visit, the nurse practitioner will treat found anal disease (if appropriate), provide any necessary referrals, and ask to be notified if a participant has any anus-related concerns in the following week. If any participant reports an abnormal outcome between Visit 1 and Visit 2, the nurse practitioner will ask the participant to return to the clinic for a DARE and any necessary treatment (and/or referral to a colorectal surgeon if treatment at the study clinic is not possible). If any participant reports an adverse event, the nurse practitioner will immediately provide necessary treatment (and/or referral to a colorectal surgeon if treatment at the study clinic is not possible). The nurse practitioner will also document the event and forward the document to the PI and co-Investigators within 24 hours. The event will then be reported to the NIH, as appropriate, to ensure safety of participants.

Study staff will be instructed to perform the ASE/ACE with gloved hands, use of lubricant, and hesitation before finger insertion to allow sphincter muscles time to relax. ASE/ACE training provided by the coordinator or educator will emphasize proper gloving technique to reduce the potential for delivery of pathogens into the anal canal or for receipt of pathogens already resident in the anal canal. In addition, the study coordinator will emphasize that a fissure at the anal os may make inserting a finger painful for the recipient. If this occurs the study staff will advise that the ASE/ACE be stopped and that the participant may need to seek medical attention. Finally, the coordinator will emphasize that palpation of a mass does not mean a person has cancer since there are a number of conditions that may create a mass.

Computer-assisted self-interviewing will be used so participants can confidentially report sensitive behaviors while minimizing feelings of shame or embarrassment discussing sexual histories.

Names will be kept separate from coded identifiers according to standards set for PHI. All computers will require a password to access files.

## Benefits

The benefit of the research to participants is that they will learn about anal conditions common among MSM and TSM who have sex with men. In addition, they will receive two examinations of their anal canal by a highly experienced nurse practitioner and thus potentially learn of an anal condition or disease and need for treatment. Thus, the risk of mild discomfort during study procedures (either physical discomfort during clinical procedures or psychological discomfort due to sensitive survey questions) are reasonable within the context of important educational and personal health information gained by the participant.

The knowledge gained by this research will benefit society by determining the viability of the ASE and ACE for persons at high risk for anal cancer and other anal conditions. This information is necessary given increasing disease incidence, the lack of infrastructure for anal cancer screening, and declining DARE use by clinicians. Data from the proposed study will propel the development of a patient-centered intervention that could be rapidly disseminated to communities with high anal cancer incidence and no currently accepted screening options; thus, the risks to participants is reasonable given the information we may learn that could help reduce anal cancer incidence.

## Adverse Event Reporting

Expected and unexpected adverse events will be reviewed by the research team on a weekly basis. We expect this study to be of minimal risk since all activities are already commonplace and routinely used in clinics globally.

## Data and Safety Monitoring Committee

The MCW Cancer Center’s Data and Safety Monitoring Committee (DSMC) will monitor this study. The DSMC will help ensure the safety of the participants by monitoring adverse outcomes throughout the study and by reviewing outcome data for possible harm. The DSMC will pay particular attention to the potential for severe anxiety in participants who believe they may have palpated a malignant disease in their anus.

The table below specifies which adverse events (AEs) and serious adverse events (SAEs) should be reported to the DSMC and when they should be reported. An SAE is defined as an adverse event that (1) results in death, (2) is life-threatening, (3) requires inpatient hospitalization or prolongation of existing hospitalization, (4) results in persistent or significant disability/incapacity, (5) results in a congenital anomaly/birth defect, or (6) is an important medical event that jeopardizes the subject or requires medical intervention to prevent one of outcomes listed above. Adverse events will be graded according to CTCAE v5.

| **Attribution** | **SAE** | | | | **AE** | | |
| --- | --- | --- | --- | --- | --- | --- | --- |
|  | **Grade 1, 2, and 3** | | **Grade 4 and 5** | | **Grade 3** | **Grade 4** | |
|  | **Expected** | **Unexpected** | **Expected** | **Unexpected** | **Unexpected** | **Expected** | **Unexpected** |
| **Possible**  **Probable**  **Definite** | **Not required to be reported** | **Within 5 calendar days^1^** | **Within 5 calendar days^1^** | **Within 5 calendar days^1^** | **Routine reporting^2^** | **Within 5 calendar days^1^** | **Within 5 calendar days^1^** |
| **Unrelated**  **Unlikely** | **Not required to be reported to DSMC** | | | | | | |
| ^1^For expedited (within 5 calendar days) DSMC reporting, the study coordinator must notify the DSMC via email including the subject ID, date of event, grade, relatedness, expectedness, and a short narrative. The event should also be entered on the Adverse Event Log.  ^2^For routine reporting, events should be entered onto the Adverse Event Log for review at the time of DSMC scheduled monitoring. | | | | | | | |

Please note that the above guidelines are for the DSMC only. SAEs also need to be reported to the IRB, and participating sites should follow their IRB’s institutional guidelines for reporting.

A running log of deviations should be kept and reported to the DSMC at time of scheduled monitoring. Any deviations requiring immediate reporting to the IRB should also be reported to the DSMC immediately.

Any other events requiring immediate reporting to the IRB (e.g., Unanticipated Problems Involving Risk to Subject or Other) should also be reported to the DSMC immediately.

A summary of the MCWCC DSMC activities are as follows:

- Review the clinical trial for data integrity and safety.
- Review any adverse events as specified above, as well as deviations.
- Submit a summary of any recommendations related to study conduct to the PI.
- Terminate the study if deemed unsafe for participants.

The committee will review the study for scheduled monitoring after completion of 100 of participant visits and at six-month intervals thereafter (or more frequently if needed) and provide recommendations on trial continuation, suspension or termination as necessary. Any available DSMC letters will be submitted to the IRB of record as required.

### **REPORTING TO COMMUNITIES**

At the time of publication of study results, public presentations to members of lay communities involved in the study will be scheduled. Study results will be published in per-reviewed journals and presented at conferences.

**References**

1. Colon-Lopez V, Shiels MS, Machin M, et al. Anal cancer risk among people with HIV infection in the United States. *J Clin Oncol.* 2018;36(1):68-75.

2. Silverberg MJ, Lau B, Justice AC, et al. Risk of anal cancer in HIV-infected and HIV-uninfected individuals in North America. *Clin Infect Dis.* 2012;54(7):1026-1034.

3. Chaturvedi AK. Beyond cervical cancer: Burden of other HPV-related cancers among men and women. *J Adolesc H.* 2010;46(4, Supplement 1):S20-S26.

4. Macaya A, Munoz-Santos C, Balaguer A, Barbera MJ. Interventions for anal canal intraepithelial neoplasia. *Cochrane Database Syst Rev.* 2012;12:CD009244.

5. Richel O, de Vries HJ, van Noesel CJ, Dijkgraaf MG, Prins JM. Comparison of imiquimod, topical fluorouracil, and electrocautery for the treatment of anal intraepithelial neoplasia in HIV-positive men who have sex with men: an open-label, randomised controlled trial. *Lancet Oncol.* 2013;14(4):346-353.

6. National Cancer Institute. NCI News Note: Multi-center anal pre-cancer treatment and cancer prevention study launched in HIV-infected persons. National Cancer Institute. <http://www.cancer.gov/news-events/press-releases/2015/anchor-trial-launch>. Published 2015. Accessed August 26, 2016.

7. D'Souza G, Wiley DJ, Li X, et al. Incidence and epidemiology of anal cancer in the Multicenter AIDS Cohort Study. *J Acquir Immune Defic Syndr.* 2008;48(4):491–499.

8. Panel on Opportunistic Infections in HIV-Infected Adults and Adolescents. Guidelines for the prevention and treatment of opportunistic infections in HIV-infected adults and adolescents: Recommendations from the Centers for Disease Control and Prevention, the National Institutes of Health, and the HIV Medicine Association of the Infectious Diseases Society of America. Available at <http://aidsinfo.nih.gov/contentfiles/lvguidelines/adult_oi.pdf>. Published 2019. Accessed August 12, 2019.

9. Darragh TM, Winkler B. Anal cancer and cervical cancer screening: Key differences. *Cancer Cytopathol.* 2011;119(1):5-19.

10. Fox P. Anal cancer screening in men who have sex with men. *Curr Opin HIV AIDS.* 2009;4(1):64-67.

11. Park IU, Palefsky JM. Evaluation and management of anal intraepithelial neoplasia in HIV-negative and HIV-positive men who have sex with men. *Curr Infect Dis Rep.* 2010;12(2):126-133.

12. New York State Department of Health-AIDS Institute: HIVQUAL-US. *HIVQual-US Annual Data Report. Retrieved on September 10, 2016 from* [*https://www.ehivqual.org/scripts/eHIVQUAL%202011%20Report%20-%20National.pdf*](https://www.ehivqual.org/scripts/eHIVQUAL%202011%20Report%20-%20National.pdf)*.* 2013.

13. Wong RK, Drossman DA, Bharucha AE, et al. The digital rectal examination: A multicenter survey of physicians' and students' perceptions and practice patterns. *Am J Gastroenterol.* 2012;107(8):1157-1163.

14. Ong J, Chen M, Temple-Smith M, et al. The inside story. Physicians' views on digital ano-rectal examination for anal cancer screening of HIV positive men who have sex with men. *J Med Screen.* 2013;20(4):188-191.

15. Ortholan C, Ramaioli A, Peiffert D, et al. Anal canal carcinoma: Early-stage tumors ≤10 mm (T1 or Tis): Therapeutic options and original pattern of local failure after radiotherapy. *Int J Radiat Oncol Biol Phys.* 2005;62(2):479-485.

16. Deshmukh AA, Zhao H, Das P, et al. Clinical and economic evaluation of treatment strategies for T1N0 anal canal cancer. *Am J Clin Oncol.* 2016:doi: 10.1097/COC.0000000000000339.

17. Chai CY, Cao HT, Awad S, Massarweh NN. Management of stage I squamous cell carcinoma of the anal canal. *JAMA surgery.* 2017.

18. Nyitray AG, Hicks JT, Hwang LY, et al. A phase II clinical study to assess the feasibility of self and partner anal examinations to detect anal canal abnormalities including anal cancer. *Sex Transm Infect.* 2018;94(2):124-130.

19. Weiss NS. Chapter 2: Diagnostic and Screening Tests: Measuring Their Ability to Predict Adverse Outcomes or Illness. In: *Clinical Epidemiology: The Study of the Outcome of Illness.* New York: Oxford University Press; 1996:9-26.

20. Darragh TM, Winkler B. Screening for anal neoplasia: anal cytology - sampling, processing and reporting. *Sex Health.* 2012;9(6):556-561.

21. Hillman RJ, Berry-Lawhorn JM, Ong JJ, et al. International Anal Neoplasia Society guidelines for the practice of digital anal rectal examination. *J Low Genit Tract Dis.* 2019.

22. Shaw JW, Johnson JA, Coons SJ. US valuation of the EQ-5D health states: Development and testing of the D1 valuation model. *Med Care.* 2005;43(3):203-220.

23. Strahan R, Gerbasi KC. Short, homogenous versions of the Marlow-Crowne Social Desirability Scale. *Journal of Clinical Epidemiology.* 1972;28:191-193.

24. Corbin JA, Strauss A. *Basics of Qualitative Research: Techniques and Procedures for Developing Grounded Theory.* 4th ed. Thousand Oaks, CA: Sage Publications; 2015.

25. Butame SA, Lawler S, Hicks JT, et al. A qualitative investigation among men who have sex with men on the acceptability of performing a self- or partner anal exam to screen for anal cancer. *Cancer causes &amp; control : CCC.* 2017;28(10):1157-1166.

26. Fairley C. Anal Cancer Examination Study. Alfred Health and The University of Melbourne. <http://www.anal.org.au/clinician/tutorials/AnalExam.htm>. Published 2012. Accessed December 4, 2017.

APPENDIX I: INFORMED CONSENT

APPENDIX II: INSTRUCTIONS FOR ASE AND ACE

APPENDIX III: PROTOCOL FOR CLINICAL SWABBING

APPENDIX IV: PROTOCOL FOR SPECIMEN PROCESSING

APPENDIX V: VISIT 1 PRE-EXAM CASI

APPENDIX VI: VISIT 1 POST-EXAM CASI

APPENDIX VII: VISIT 2 PRE-EXAM CASI

APPENDIX VIII: VISIT 2 POST-EXAM CASI

APPENDIX IX: SEMI-STRUCTURED QUALITATIVE INTERVIEW GUIDES

True Negative

True Positive

False Positive

False Negative

APPENDIX X: SCRIPTS

Setting an appointment

Clinic Session: Anal Anatomy, Pathology, and ASE/ACE Procedures

APPENDIX X1: COORDINATOR FORMS

Clinic Activity Checklist

Abnormal ASE Finding Detail

Abnormal ACE Finding Detail

APPENDIX X11: CLINICIAN FORMS

DARE and Swabbing

APPENDIX X111: PARTICIPANT FORMS

ASE Finding

ACE Finding

Anal Medical History

APPENDIX XIV: KYOTO MODEL (INSERT Kyoto sales flyer)
